# Development of VUMAT and VUHARD Subroutines for Simulating the Dynamic Mechanical Properties of Additively Manufactured Parts

**DOI:** 10.3390/ma15010372

**Published:** 2022-01-05

**Authors:** Amos Muiruri, Maina Maringa, Willie du Preez

**Affiliations:** 1 Department of Mechanical and Mechatronics Engineering, Central University of Technology, Bloemfontein 9301, South Africa; mmaringa@cut.ac.za; 2 Centre for Rapid Prototyping and Manufacturing, Faculty of Engineering, Built Environment and Information Technology, Central University of Technology, Bloemfontein 9301, South Africa; wdupreez@cut.ac.za

**Keywords:** additive manufacturing, DMLS, Ti6Al4V (ELI), modelling and simulations, VUMAT, VUHARD

## Abstract

Numerical modelling and simulation can be useful tools in qualification of additive manufactured parts for use in demanding structural applications. The use of these tools in predicting the mechanical properties and field performance of additive manufactured parts can be of great advantage. Modelling and simulation of non-linear material behaviour requires development and implementation of constitutive models in finite element analysis software. This paper documents the implementation and verification process of a microstructure-variable based model for DMLS Ti6Al4V (ELI) in two separate ABAQUS/Explicit subroutines, VUMAT and VUHARD, available for defining the yield surface and plastic deformation of materials. The verification process of the implemented subroutines was conducted for single and multiple element tests with varying prescribed loading conditions. The simulation results obtained were then compared with the analytical solutions at the same conditions of strain rates and temperatures. This comparison showed that both developed subroutines were accurate in predicting the flow stress of various forms of DMLS Ti6Al4V (ELI) under different conditions of strain rates and temperatures.

## 1. Introduction

Additive manufacturing (AM) technologies make it possible to design and fabricate lightweight metallic structural parts in real time. This is plausible, as components from 3D CAD models can be printed or produced directly using an electron beam source for electron beam melting (EBM) [1] or a laser source for selective laser melting (SLM)/direct metal laser sintering (DMLS) [1] on a powder bed table. Structural parts with simple to complex geometry in 3D CAD models can be fabricated via AM, since the technology offers great flexibility in design and manufacturing. Complex components with hollows and undercuts, such as turbine blades with internal cooling channels, are now being produced by AM [2]. Other complex components produced by this technology are patient-specific bio-implants such as dental prostheses and orthopaedic implants [3]. Therefore, the AM-produced components possess increased applications in the biomedical and aerospace industries.

As AM revolutionises manufacturing of functional components, much attention has focussed not only on accumulating exhaustive knowledge on the process itself, but also on resultant microstructures and mechanical properties of the manufactured parts. Steel, as the most common engineering structural material, is manifestly of high interest for AM. The grades of steel presently being produced by EBM and DMLS processes include maraging steel (18Ni300), stainless steels (17-4 PH, 15-5 PH, 321, 347, SAE 316L), and AISI 4340 steel [4]. The number of aluminium alloys produced by AM is still limited. The possible reason for this is that it is easy to machine aluminium, and furthermore the cost of aluminium parts is comparably low [5]. Therefore, the use of AM in the production of aluminium parts is often deemed as of low commercial benefit [5,6]. Aluminium alloys are also regarded as challenging when it comes to processing by AM. For instance, aluminium powder has been shown to have lower absorptivity of the laser, conventionally used in SLM/DMLS [7]. More details on challenges associated with AM of aluminium alloys can be found in [8]. The aluminium alloys that have shown promising results in terms of processability by SLM are generally AlSi10Mg, which has received the most attention, and hypo-eutectic AlSi12 [7,8].

Titanium alloys parts are produced widely by the AM process. This is mainly because of their broad industrial applications in high performance structural parts. Usually, these parts have high machining costs and a long lead time when produced by traditional manufacturing routes [6]. Titanium alloys are also very expensive; thus, AM offers a unique opportunity of reducing the cost by reducing the requirements for raw materials as well as the extent of required post-machining operations. Therefore, many business cases in the aerospace and biomedical industry exist for AM of titanium alloys due to attendant substantial cost benefits. The Ti6Al4V parts produced by AM have been investigated widely by material scientists and engineers, and many components of AM are produced for commercial purposes worldwide [9].

A fine-grained microstructure, commonly referred to as acicular (α’) martensitic microstructure, is usually obtained for DMLS Ti6Al4V (ELI) [10,11]. This is normally due to the thermal cycle experienced by the alloy during the building process [12]. This thermal cycle involves rapid heating above the melting temperature by an electron or laser beam. Rapid solidification of the molten material after the heat source has moved on then follows. The melted and solidified layers then experience numerous rapid re-heating and re-cooling processes when the material volume element in the top current layer is exposed to heat [6,12]. Thus, the DMLS process leads to the formation of meta-stable microstructures with non-equilibrium composition of resulting phases such as the acicular (α’) martensitic microstructure in the case of Ti6Al4V (ELI). This type of microstructure is characterised by high strength and low ductility [13]. However, improved ductility with reduction in strength is realised for DMLS Ti6Al4V (ELI) following successive heat treatments involving the transformation of the non-equilibrium α’-phase into an equilibrium mixture of (α + β) phases.

Numerous studies have applied one stage and two-stage heat treatment cycles at different temperatures on the as-built parts with the aim of optimising the mechanical properties of AM alloys to satisfy industrial requirements [10,11,13,14,15]. Different heat treatment cycles yield different mechanical properties of strength, ductility, and toughness, based on the resulting microstructure. The most critical aspects of the microstructure that influence the properties of alloys are the grain morphology and size, crystallographic texture, and defects which are largely dislocations [16]. Other external factors, such as the prevailing level of strain rate and temperature, affect the yield stress and deformation properties of metals and alloys. All these factors must be considered in the development of energy-efficient structural components. Conventionally, many experiments are required to study the effect of these factors on the mechanical performance of materials. Production and post processing of AM test samples and experimental tests to study the mechanical properties for a wide range of external state variables are costly and time consuming. Modelling and simulations are alternative powerful methods that can be used to this end.

Large parts of most modern industries throughout the production chain are supported by modelling and numerical simulation [17]. A manufacturing chain simulation can be used to compute the progressive effects of different processes along the production line. By pursuing the material state throughout the entire production chain from the initial state of the material, the mechanical properties of the final product can be predicted realistically [18]. The numerical modelling of the metal AM processes, resulting microstructures, and subsequent mechanical properties can be used to support experimental studies in the quest for achieving optimally produced parts with tailored mechanical properties. Numerical modelling and simulation can be useful to predict mechanical properties of materials, such as yield stresses and flow stresses, in those circumstances where actual experiments are too costly or even difficult to perform [18,19].

Various researchers have attempted to model and simulate the AM process and resulting microstructures. The finite element modelling (FEM) of an AM process to optimise the process parameters and therefore minimise residual stresses and distortion was reported in [20]. In this research work, the FEM software ABAQUS was employed to simulate an AM process, as it provides an interface allowing the user to define input data, such as element and heat input, that are a function of both position and time to achieve the process simulation of 3D parts. Marques et al. [21] used numerical analysis of the SLM process to assess the effects of laser scanning strategies on the residual stresses generated in the fabrication of Ti6Al4V parts. Chen et al. [22] used a 3D finite element model to simulate thermal behaviour in the SLM process and further investigated the effects of laser beam power and laser scanning speed on the thermal behaviour and solidification of SLM-produced high strength tool steel. Using the cellular automation (CA) method to describe grain growth, Zinoviev et al. [23] developed a two-dimensional numerical model to simulate the evolution of grain structure observed during the laser additive manufacturing process. Yang et al. [24] used a cellular automation model to simulate microstructural solidification and solid-state phase transformation of the Ti6Al4V alloy under various spatially variable thermal cycles of the SLM process. In their work, the morphology and size of β-grains and martensite simulated by the model agreed well with experimental results. Artificial neutral network (ANN) models have also been utilised to optimise AM process parameters and predict the mechanical behaviour of 3D-printed parts [25,26]. These types of models depend on huge amounts of experimental data for model-training purposes, and constitutive mathematical expressions describing the material properties are not required. This last bit makes it difficult to use ANN models to develop numerical models for predicting the mechanical properties of AM parts.

This study focuses on developing a numerical model based on the dominant mechanisms of deformation of microstructures of AM Ti6Al4V (ELI), over a broad-spectrum of temperatures and strain rates. To carry out modelling and simulation of a non-linear analysis considering large displacements and elastoplastic material behaviour, user-defined material models are required. This is so in the present case where in-built models in finite element analysis (FEA) packages are inadequate. The commercial package of ABAQUS provides user interfaces via a FORTRAN compiler linking to the main program to allow the user to formulate and incorporate user-defined material models [27].

Therefore, the developed, calibrated, and validated microstructure- and dislocation- based analytical constitutive model reported in the authors’ previous work in [28,29] is implemented in this study as a user material subroutine in ABAQUS using VUMAT and VUHARD subroutines. The VUHARD subroutine is used to describe the plastic deformation of a material, while the VUMAT subroutine describes the complete (elastic-plastic) behaviour of a material. Both subroutines use an explicit integration scheme (forward Euler method), which calculates the state of the variables from the model at the current time [27]. Implementation of the VUHARD subroutine is easy and straightforward and requires only the constitutive model and its derivatives. Implementation of the VUMAT subroutine, on the other hand, is involved and requires a clear understanding of the von Mises yield criterion and the radial return algorithm method used to evaluate the increment of state variables. A summary of the von Mises yield criterion and the radial return algorithm used in most FEA programs is presented in Appendix A.

The codes developed in the present work for each of the two ABAQUS user subroutines are presented here as Supplementary Material. The implemented subroutines were verified using single and multiple element tests with varying prescribed loading. The simulation results obtained were then compared with the analytical solutions developed and reported elsewhere by the author [28] and showed good accuracy for both the developed VUMAT and VUHARD subroutines applied to high-strain-rate numerical simulations.

## 2. A Microstructure and Dislocation-Based Model for DMLS Ti6Al4V (ELI)

This section presents an analytical model that uses the critical microstructural parameters of various forms of DMLS Ti6Al4V (ELI) to predict the mechanical properties of the alloy over a wide range of strain rate and temperature. Details on formulation of the model were presented in the authors’ previous work published in [29]. The total flow stress of a given microstructure of DMLS Ti6Al4V (ELI) is dependent on internal and external state variables. The internal state variables include the average grain size and the density of dislocations, while the external state variables are field variables of strain, strain rate, and temperature. The incorporation of mechanical threshold stress (MTS), the Hall–Petch relationship, the Taylor strain hardening law, and viscous drag effects at a high strain rate were used in [29] to describe the yielding and flow stress of DMLS Ti6Al4V (ELI) microstructures and a wide range of field variables. The total flow stress developed this way is of the following form:

(1) σ=(μ(T)μ0)σo(1−((kbTg0iμ(T)b3)ln ε˙oε˙)1q)1p+ζ.(1−exp(−χ .ε˙p))+KH−Pt +αμ(T)bM(hk2 (1−exp(−k2εp ))+ρoexp(−k2εp))12

The first part of the foregoing expression accounts for the MTS where the symbol $\sigma^{o}$ is the mechanical threshold stress or the value of the thermal stress at 0 K, *μ* is the shear modulus dependent on temperature T, and $\mu_{o}$ is the shear modulus at absolute zero temperature (0 K). The symbols $k_{b}$, b, $g_{oi}$, ${\dot{\varepsilon }}_{o}$, and $\dot{\varepsilon }$ are the Boltzmann’s constant, the Burgers vector, the material constant, the reference strain rate, and the testing strain rate, respectively. The constants p and q are model fitting parameters. The second part of Equation (1) describes the upsurge of flow stress as the plastic strain rate (${\dot{\varepsilon }}_{p}$) increases because of viscous drag effects, and the symbols ζ and χ in it are viscous drag stress calibration parameters. The third part of this equation represents the inverse relationship between the average thickness of α-laths, t, and the yield stress of a material, where $K_{H-P}$ is the Hall–Petch constant. The fourth and last part of this equation is the integrated Taylor hardening law which represents the accumulation of dislocations with increase in plastic strain. In this part of the equation, the symbol α is a dimensionless parameter of magnitude ranging from 0.2 to 0.4 for different materials. The parameter M is the Taylor factor which relates the shear flow stress τ of a single crystal to the uniaxial flow stress σ of a polycrystal. The constants h and $k_{2}$ stand for the coefficient of dislocation accumulation and annihilation, respectively, while $\rho_{0}$ is the total initial dislocation density in a particular material’s microstructure.

## 3. Materials and Methods

### 3.1. Materials

The calibration and validation of the flow stress model in Equation (1) were reported in [29] and required material properties obtained from analysis of microstructures and experimental tests. Cylindrical rods with a height of 80 mm and diameter 6 mm were produced from Ti6Al4V (ELI) (grade 23) powder by the DMLS process using an EOS M280 machine. The chemical composition (in wt.%) of this powder as provided by the suppliers, TLS Technik GmbH (Bitterfeld-Wolfen, Germany), is Al: 6.34, V: 3.944, Fe: 0.25, O: 0.082, C: 0.006, N: 0.006, H: 0.00, and the balance Ti.

**Figure 1:**
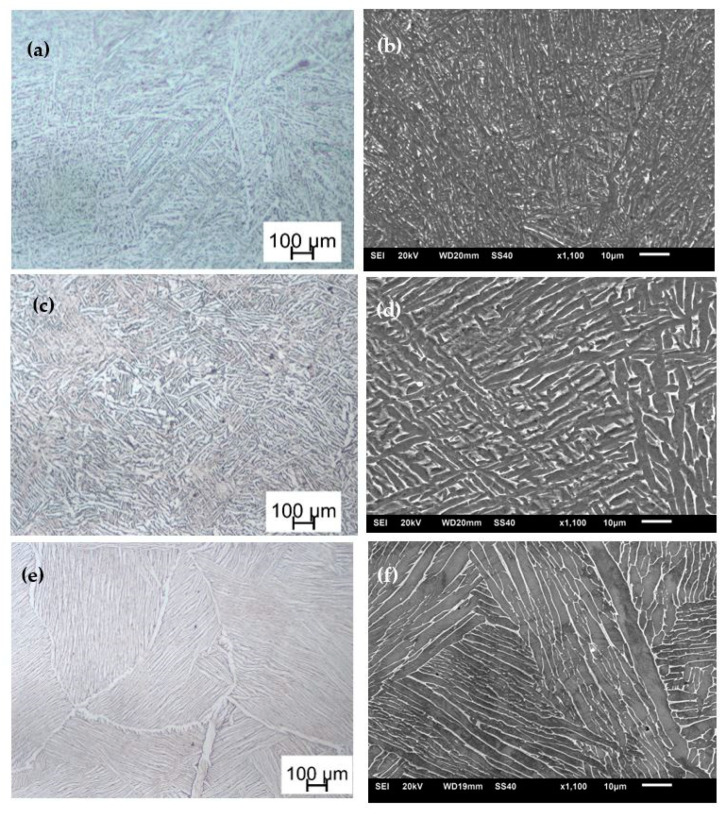
(**a**,**c**,**e**) Optical images and (**b**,**d**,**f**) secondary electron images of (**a**,**b**) samples C, (**c**,**d**) samples D, and (**e**,**f**) samples E, respectively.

These rods were first stress relieved at a temperature of 650 °C for 3 h and then furnace cooled to room temperature. The stress relieved rods were then subdivided into three groups designated hereinafter as samples C, D, and E for different heat treatment processes. The samples designated C were heat-treated at 800 °C for 2.5 h and then furnace-cooled to room temperature. The samples designated D were duplex-annealed at 940 °C for 2 h and then furnace-cooled, followed by heat treatment at 750 °C for 2 h and then finally furnace-cooled. The samples designated E were heat treated above the β-transformation temperature at 1020 °C for 2.5 h and then furnace-cooled to room temperature. The optical images and secondary electron images of the microstructures obtained after these different heat treatment strategies are shown in Figure 1a–f.

The detailed description of these microstructures can be found in [10,28,30]. The average α-grain size in each of these microstructures was determined by the line intercept method and results presented in [10]. The total initial dislocation density in these heat-treated samples was also determined by X-ray diffraction peak broadening analysis and the results reported in [30]. As seen in Equation (1), the average grain size and the initial dislocation density are the two critical internal state variables in any given microstructure of DMLS Ti6Al4V (ELI) required to describe the flow stress. These important numerical model input parameters for different forms of DMLS Ti6Al4V (ELI) are shown in Table 1.

### 3.2. Experimental Tests

The flow stress curves of samples C, D, and E over a wide range of strain rates and temperatures were obtained using the Kolsky bar commonly known as the split-Hopkinson pressure bar (SHPB). The tests were conducted at strain rates of 750, 1500, and 2450 s^−1^ and the tests at each strain rate carried out at the three different temperatures of 25, 200, and 500 °C. The results obtained in these tests were reported in [28], while the calibration and validation of the microstructural sensitive model presented in Section 2 using flow curves in [28] were reported in [29]. A summary of the model-calibrated fitting parameters obtained from this analytical modelling and other physical constants used and obtained from literature is presented in Table 2.

## 4. Implementation of Subroutines

### 4.1. Implementation of the Microstructure-Based Constitutive Flow Stress Model as a VUHARD Subroutine

Implementation of the VUHARD user subroutine for the microstructure-based constitutive formulation of flow stress was done by first obtaining the derivatives of this constitutive model with respect to appropriate variables. In this constitutive model, presented here as Equation (1), the total flow stress is seen to be dependent on three external state variables ($\varepsilon_{p},\;{\dot{\varepsilon }}_{p}$, and  T), whilst the internal state variables can be obtained from the microstructure. The temperature-dependence of the constitutive model was taken care of by the decrease in shear modulus (μ) with an increase in temperature using the equation for shear modulus shown in Table 2.

Therefore, in this study, implementation of the VUHARD subroutine for the constitutive model required only two analytical derivatives of $\left(D\;1=\partial \sigma /\partial \;\varepsilon_{p}\right)$ and $\left(D\;2=\partial \sigma /\partial {\dot{\varepsilon }}_{p}\right),$ which were then evaluated numerically to allow quadratic convergence. These derivatives represented the change in the calculated total flow stress for the inputs of equivalent plastic strain rate and equivalent plastic strain. The term “equivalent” is used here to denote the scalar quantity of strain and strain rate obtained from the transformation of the strain tensor using the von Mises criterion (presented and discussed in Appendix A), which is the common criterion used in ABAQUS. The partial derivative $\partial \bar{\sigma^{n\;+1}}/\partial \bar{\varepsilon_{p}{}^{n+1}}$ was determined numerically from the following equation:

(2) $$\begin{array}{ll} \frac{\partial \bar{\sigma^{n+1}}}{\partial \bar{\varepsilon_{p}^{n+1}}}=\frac{\alpha \mu \left(T\right)bM}{2} & {\left(\frac{h}{k_{2}}\;\left(1-\exp\left(-k_{2}\bar{\varepsilon_{p}^{n+1}}\right)\right)+\rho_{o}\exp\left(-k_{2}\bar{\varepsilon_{p}^{n+1}}\right)\right)}^{-1/2}.(h\;exp\left(-k_{2}\bar{\varepsilon_{p}^{n+1}}\right)\\ & -\rho_{o}k_{2}exp\left(-k_{2}\bar{\varepsilon_{p}^{n+1}}\right))\; \end{array}$$

Similarly, the other derivative, with respect to plastic strain rate, $\bar{{\dot{\varepsilon }}_{p}^{n+1}},$ was evaluated from the following equation:

(3) $$\begin{array}{ll} \frac{\partial \bar{\sigma^{n+1}}}{\partial \bar{{\dot{\varepsilon }}_{p}^{n+1}}}\left(\frac{\mu \left(T\right)}{\mu_{0}}\right). & \frac{\sigma_{o}k_{b}T^{n+1}}{pq\bar{{\dot{\varepsilon }}_{p}^{n+1}}G^{n+1}b^{3}}{\left(1-{\left(\frac{k_{b}T^{n+1}}{g_{0i}G^{n+1}b^{3}}ln\left(\frac{{\dot{\varepsilon }}_{o}}{\bar{{\dot{\varepsilon }}_{p}^{n+1}}}\right)\right)}^{\frac{1}{q}}\right)}^{\frac{1-p}{p}}.{\left(\frac{k_{b}T^{n+1}}{g_{0i}G^{n+1}b^{3}}ln\left(\frac{{\dot{\varepsilon }}_{o}}{\bar{{\dot{\varepsilon }}_{p}^{n+1}}}\right)\right)}^{\frac{1-q}{q}}\\ & +\alpha exp\left(-\chi .\bar{{\dot{\varepsilon }}_{p}^{n+1}}\right) \end{array}$$

The VUHARD user subroutine written in this work is included in this paper as Supplementary Material in S1. The coding for this subroutine was generated using FORTRAN 90 and saved in a *.for* file, formatted to be compatible with ABAQUS 2020. The first algorithm for the subroutine was used to generate the user material properties described as user material constants. A total of 17 user material constants, derived from Equation (1) and listed in Table 3, were generated.

These excluded the material density, elastic modulus, and Poisson’s ratio that were provided separately in ABAQUS/CAE. The flow stress component that is dependent on the average thickness of α-laths (t) was computed based on the Hall–Petch relationship and was input into the subroutine as an average variable of α-lath thickness (a variable that depends on the form of the alloy).

The previous and current values of equivalent plastic strain in a step were defined as old and new solution-dependent variables (SDV1), respectively. Thus, the increment of plastic strain was obtained as the difference between these two states. The equivalent plastic strain rate component of Equations (1) and (3) was expressed from the increment in equivalent plastic strain ($\Delta \bar{\varepsilon_{p}}$) for a time increment size (Δt) as follows:

(4) $$\bar{{\dot{\varepsilon }}_{p}}=\frac{\Delta \bar{\varepsilon_{p}}}{\Delta t}$$

Equations (1)–(3) were evaluated at given material points and arrays containing values of equivalent yield stress, plastic strain, and strain rate, generated at related material points.

### 4.2. Implementation of the Microstructure-Based Constitutive Flow Stress Model as a VUMAT Subroutine

**Figure 2:**
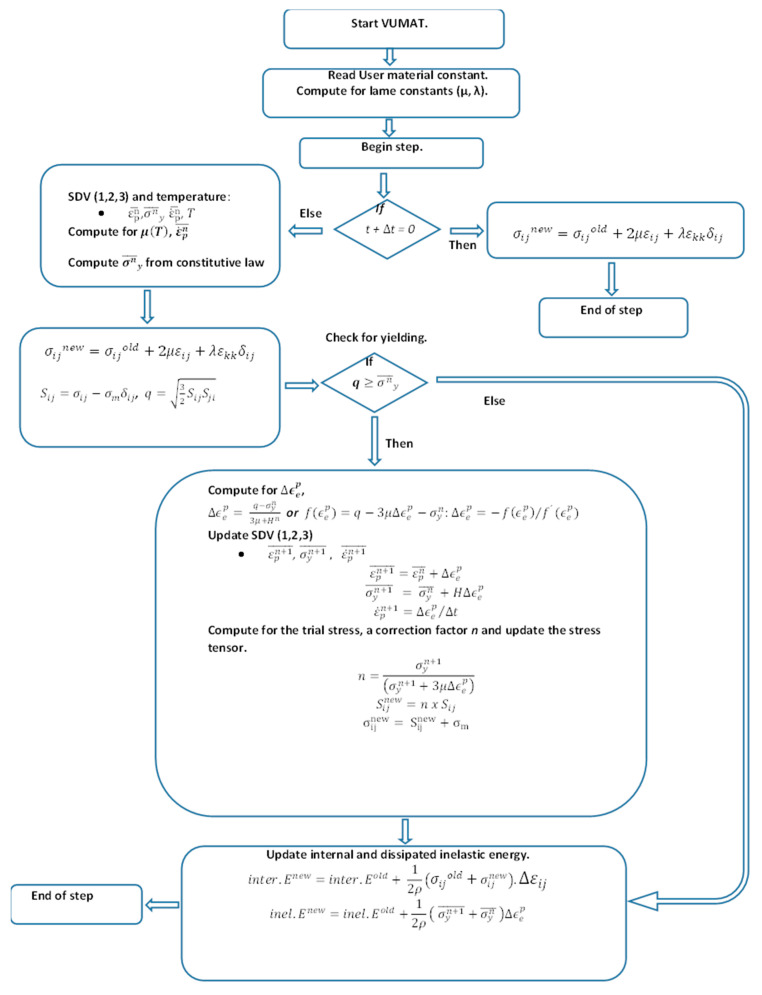
Algorithm developed in the present work to solve for equivalent plastic strain increments and updating of the related stress tensors.

To make it easier to implement the microstructure- and dislocation-based constitutive model as a VUMAT user material subroutine, the implementation algorithm was written following the logic of the flow chart shown in Figure 2. Coding for this subroutine was based on the theory of plasticity and radial return mapping presented in Appendix A.

Similar to VUHARD, the coding for VUMAT was generated in FORTRAN 90 and saved as a .*for* file for execution in ABAQUS 2020. The codes written are presented in S2 in the Supplementary File. The second block in the algorithm was used to obtain the model constants of the material. Both elastic and plastic properties are required in this subroutine’s main program, which is different from VUHARD where only the plastic properties are provided in the main program and the elastic properties are provided in ABAQUS/CAE. Thus, the 17 material constants from Equation (1) in addition to the material’s elastic modulus (Ε) and Poisson’s ratio (ν) were used for this subroutine. These material constants are presented in Table 3. The two Lamé’s constants (μ and λ) were computed from the elastic modulus (Ε) and Poisson’s ratio (ν) using the following two expressions for isotropic hardening:

(5) $$\mu =\frac{E}{2\left(1+\nu \right)}\;\mathrm{and}\;\lambda =\frac{\nu E}{\left(1-2\nu \right)\left(1+\nu \right)}$$

The principal components of the stress tensor were first obtained at the reference time t+Δt=0 from the following trace of principal strain increments:

(6) $$trace\left(\Delta \varepsilon \right)=\Delta \varepsilon_{11}+\Delta \varepsilon_{22}+\Delta \varepsilon_{33}\;$$

At time (t+Δt>0), the VUMAT algorithm was split in three parts. In the first part, the values of equivalent plastic strain ($\bar{\varepsilon_{p}^{n}}$), equivalent plastic strain rate ($\bar{{\dot{\varepsilon }}_{p}^{n}}$), and yield stress $\sigma_{y}^{n}$ (given by the constitutive model at $\bar{\varepsilon_{p}^{n}}$ and $\bar{{\dot{\varepsilon }}_{p}^{n}}$ and at a prescribed test temperature (T)) at the beginning of the increments at each integration point were defined as SDV1, SDV2, and SDV3, respectively. The values of shear modulus used in the constitutive model to define the flow stress were computed at prescribed test temperatures before obtaining the value of flow stress in this part.

In the second part, the theory of plasticity presented in Appendix A was used to compute equivalent von Mises trial stresses *q*. The third part aimed at comparing the values of equivalent von Mises trial stresses obtained with the values of yield stress (from the constitutive model given by Equation (1)) obtained at the beginning of each increment such that:

▪ If $q<\sigma_{y}^{n}$, then the state response was elastic, and the radial return algorithm that is inherent in the subroutine to correct the plastic response was skipped. The stress tensor and related deviatoric stress remain unchanged at any value of *q*. Therefore, the last step of the algorithm to compute internal energy and dissipation of inelastic energy was initiated. It is important to note that internal energy, abbreviated as *ALLIE* in ABAQUS, is the sum of the recoverable elastic strain energy and the energy dissipated through plastic deformation [27]. Since there was no plastic deformation, the inelastic energy remained zero, and the internal energy only included the elastic strain energy component.
▪ If $q\;\geq \sigma_{y}^{n}$, then the stress state was outside the yield surface and was plastic. Thus, the radial return mapping previously described in Appendix A was initiated to compute the equivalent plastic strain and therefore return the predicted stress back to the expanded yield surface of the material. At this stage, strain hardening was expected since the loading was beyond the elastic limit.

The final part of this algorithm updated the SDVs and computed the new internal energy and inelastic dissipated energy. The updated SDV1, SDV2, and SDV3 at each stage of computation, which were now the new values of equivalent plastic strain $\bar{\varepsilon_{p}^{n+1}}$, the new equivalent plastic strain rate $\bar{{\dot{\varepsilon }}_{p}^{n+1}}$, and the new yield stress $\sigma_{y}^{n+1},$ were stored and then used in the next increment as current values. For linear isotropic materials undergoing small strains, the strain energy is of the following form [27]:

(7) $$U=\frac{1}{2}\sum_{i,j=1}^{3}\sigma_{ij}\varepsilon_{ij}$$

From Equation (7), the total new specific internal energy ($inter.E^{new}$) and specific inelastic dissipation energy ($inel.E^{new}$) were expressed as

(8) $$inter.E^{new}=inter.E^{old}+\frac{1}{2\rho }\left(\sigma_{ij}{}^{old}+\sigma_{ij}^{new}\right).\Delta \varepsilon_{ij}$$

(9) $$inel.E^{new}=inel.E^{old}+\frac{1}{2\rho }\left(\;\bar{\sigma_{y}^{n+1}}+\bar{\sigma_{y}^{n}}\;\right)\Delta \epsilon_{e}^{p}$$

## 5. Results and Discussions

The microstructure- and dislocation-based constitutive model implemented as VUHARD and VUMAT subroutines was verified by determining the closeness of the results of simulation using ABAQUS to the analytical solutions based on Equation (1). The user material constants in this equation were those that were used to fit the equation to experimental results for samples C, D, and E, which have been presented in Table 1 and Table 2. Various benchmark tests were used here ranging from single element tests to multiple element tests.

### 5.1. The Single Element Tests

**Figure 3:**
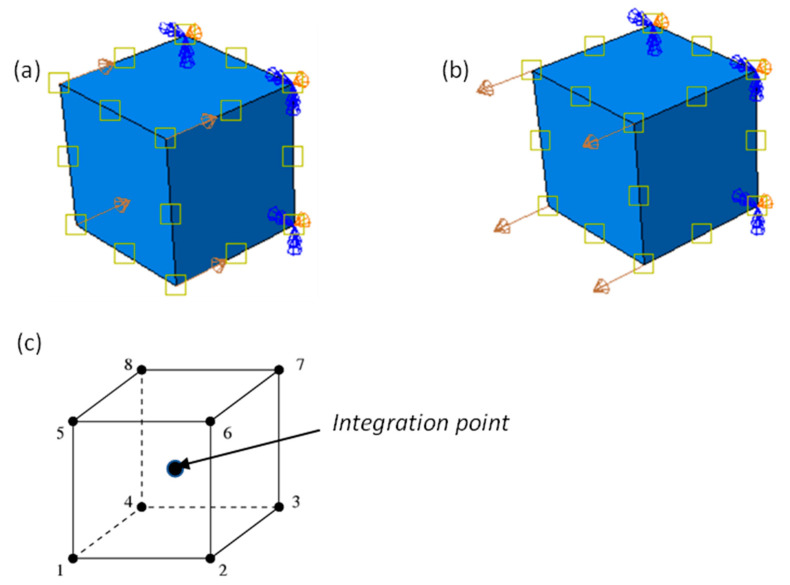
(**a**,**b**) A single element model with boundary conditions and (**c**) the 8-node linear brick element.

The verification process adopted in the present research was to first test the material model developed in ABAQUS using single eight-node linear brick element tests. This is an easy and practical technique to examine the sensitivity and accuracy of an element to external loading. Here, the single eight-node linear brick element tests were performed using the prescribed velocity loading conditions both in compression and tension, as shown in Figure 3a,b, respectively.

The roller support condition to restrain the surface in a direction parallel to the loading direction was applied to the four nodes of the surface opposite to the surface onto which load was applied. The dimensions of the cube were arbitrarily taken as (10 × 10 × 10) mm and the cube meshed using the eight-node linear brick C3D8R element shown in Figure 3c, with reduced integration and hourglass control settings. This type of mesh is simple and appropriate for the cubic element models of components with regular straight-edged shapes.

The reduced integration elements normally have a single integration point located at the element’s centroid rather than full integrated element types and hence give rise to reduced computation time. However, reduced integration elements tend to be too flexible, since they suffer from their own numerical problem called hourglassing. Hourglassing arises where elements are unable to resist bending and hence cannot store strain energy for bending deformation. In ABAQUS hourglass control, a small amount of artificial stiffness is introduced in elements to limit the propagation of the hourglass phenomenon [27].

**Figure 4:**
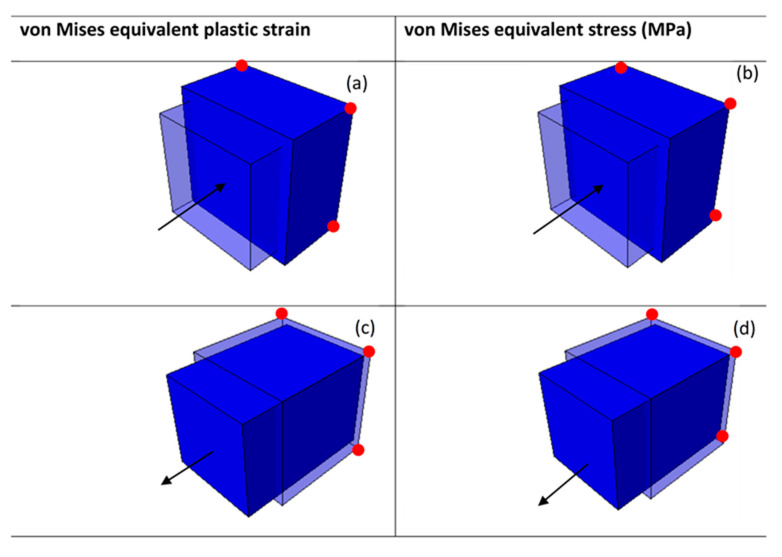
A typical VUHARD deformed 8-node linear brick element at a temperature of 298 K and velocity of 4 m/s in (**a**,**b**) compression and (**c**,**d**) tension for sample type C. The arrows show the loading direction, while the red dots are the roller supports.

**Figure 5:**
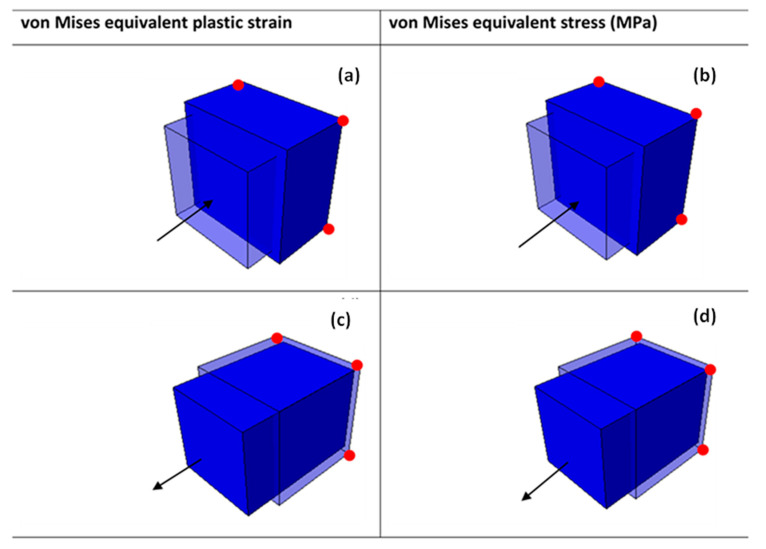
A typical VUMAT deformed 8-node linear brick element at a temperature of 298 K and velocity of 4 m/s in (**a**,**b**) compression and (**c**,**d**) tension for sample type C. The arrows show the loading direction, while the red dots are the roller supports.

Three different prescribed instantaneous velocities of 0.1 m/s, 1 m/s, and 4 m/s were then applied onto the cube in both compression and tension. These different velocities were used to generate high equivalent plastic strain rates of approximately 10 s^−1^, 123 s^−1^, and 350 s^−1^. A typical deformed eight-node linear brick element at 4 m/s is shown in Figure 4 and Figure 5. The shapes plotted in these figures are for superimposed undeformed and deformed shapes of the element under compressive and tensile load. The values of equivalent plastic strain are normally expressed as PEEQ (this abbreviation stands for von Mises equivalent plastic strain in ABAQUS) in VUHARD, as shown in Figure 4a,c, while in VUMAT, they are allocated space as the solution-dependent variable, SDV1, seen in Figure 5a,c.

The equivalent plastic strain and equivalent von Mises stresses were obtained at the integration/Gauss point shown in Figure 3c. Lateral expansion of the cube is realised in the case of a compression test from the surface where load was applied to the surface under the constraints, as shown in Figure 4a,b and Figure 5a,b. To the contrary and as expected, the deformed tensile element is characterised by lateral reduction in the cross-sectional area, as shown in Figure 4c,d and Figure 5c,d. The values of equivalent von Mises stress and equivalent plastic strain shown in the keys of Figure 4 and Figure 5 were obtained from the field output.

**Figure 6:**
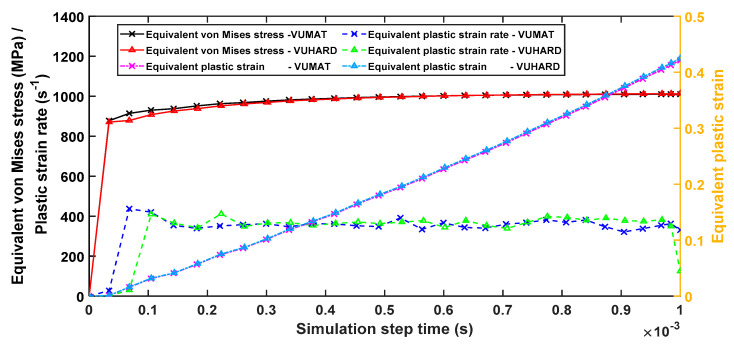
The equivalent von Mises stress, plastic strain, and strain rate against simulation time history generated in VUMAT and VUHARD subroutines developed here for a test velocity of 4 m/s and a temperature of 298 K.

With dynamic explicit integration of instantaneous imposed velocities, step times are important since long step times could cause excessive deformation of elements. In some instances, the opposite faces may cross over each other (in compression) causing the job to exit with an error, whilst for very small step times, the elements may not reach the plastic deformation state. For single element tests, the dynamic explicit integration option with total step times (*t*) of 0.03 s, 0.003 s, and 0.001 s for velocities of 0.1 m/s, 1 m/s, and 4 m/s, respectively, were found to give sufficient deformation without excessive distortion of the element. For velocities beyond 4 m/s for this kind of numerical model, the simulation aborted even for very small step times due to excessive deformation of the model. Figure 6 shows the change in the values of equivalent von Mises stresses, plastic strains, and plastic strain rates for samples C with increasing simulation time in VUHARD and VUMAT for a velocity of 4 m/s. In both subroutines, the plastic strain is initially zero (during elastic deformation) before increasing uniformly to a maximum value, and therefore the rate of deformation (plastic strain rate) is constant during this period, as seen in Figure 6. Both VUMAT and VUHARD show fairly similar values of average plastic strain rate of approximately 350 s^−1^ in the figure. The equivalent plastic strain and flow stress curves generated from the two subroutines also coincide for the entire and a large part of the simulation step time, respectively.

**Figure 7:**
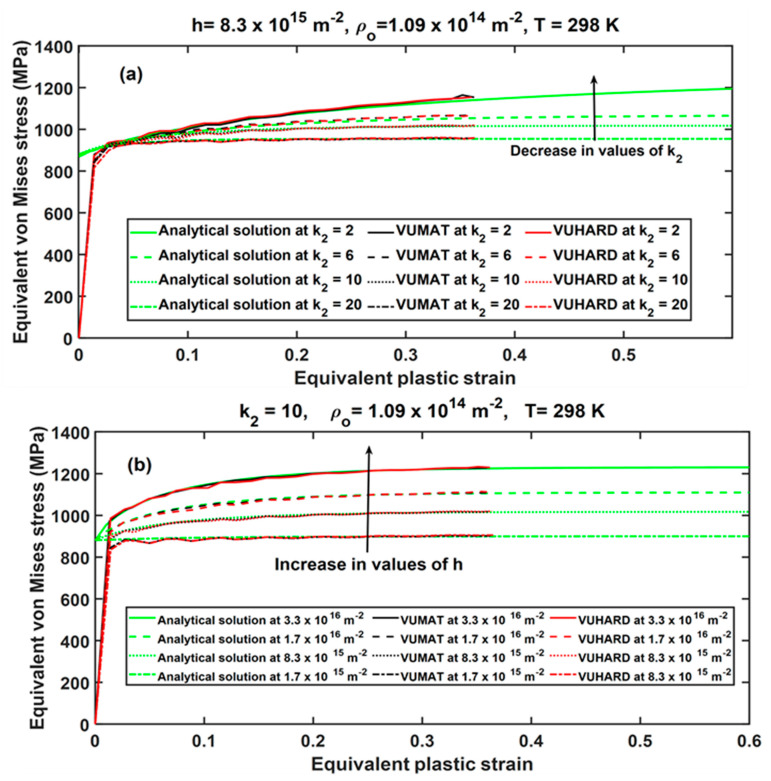
The effects of model parameters (**a**) $k_{2}$ and (**b**) h on the numerical solutions derived from VUMAT and VUHARD subroutines and compared with the analytical solution at the same conditions of 350 s^−1^ and 298 K.

**Figure 8:**
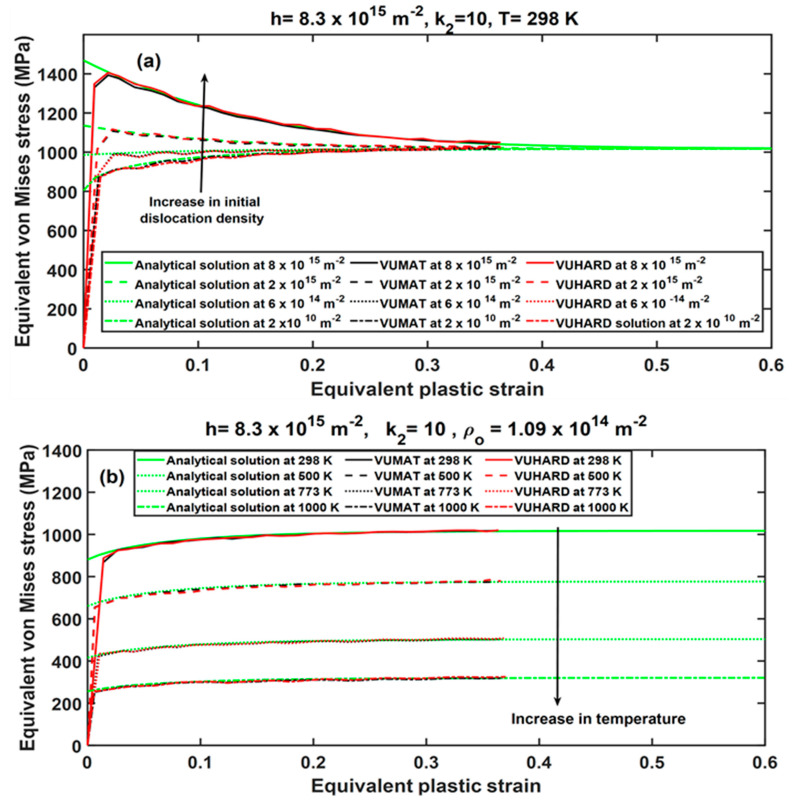
The effects of (**a**) initial dislocation density ($\rho_{o}$) and (**b**) temperature on the numerical solutions derived from VUMAT and VUHARD subroutines, compared with the analytical solutions at the same average strain rate of 350 s^−1^.

As discussed in the authors’ previous work reported in [29], there are four critical parameters of the constitutive model implemented in the subroutines in this research that influence the shape of the flow stress curve, namely h, $k_{2}$, $\rho_{o}$, and T. The sensitivity of these critical parameters in the numerical models for samples C developed here was investigated at a velocity of 4 m/s, and the results were compared with those of the analytical solutions at the same conditions for both cases. These results are presented in Figure 7 and Figure 8.

As seen in these figures, the results from the numerical models derived in VUMAT and VUHARD, and those from the analytical solutions developed here, coincide for a large part of the flow curves. The opposing effect of parameters h and $k_{2}$ is well articulated in the numerical model where the flow stress increases with decreasing values of the parameter $k_{2}$, whereas it increases with increasing values of the parameter h, as seen in Figure 7. Similar observations were reported in [29]. Figure 8 shows the effects of initial dislocation density and temperature on the numerical solutions obtained from the VUMAT and VUHARD subroutines developed here and the analytical solutions, all at the same strain rate of 350 s^−1^. It is clear from Figure 8a that for very high initial dislocation densities, the flow stress curve is characterised by high initial peak stresses followed by a decrease in flow stress. For very low initial dislocation densities, the peak stresses are lower, and the material experiences strain hardening. Similar observations were noted in [29], and the theory behind this phenomenon was presented.

**Figure 9:**
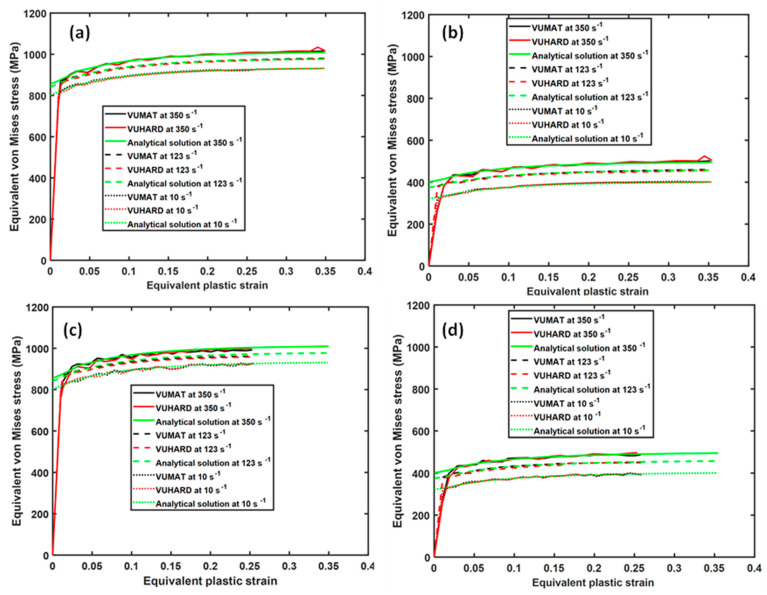
Comparison between the analytical and numerical solutions in VUMAT and VUHARD at three different test velocities and temperatures of (**a,c**) 298 K and (**b**,**d**) 773 K for an 8-node linear brick element in (**a,b**) compression and (**c**,**d**) tension for samples C.

The effects of simulation temperatures on the flow stress curve are very conspicuous in Figure 8b, where the values of flow stress are seen to decrease with increase in temperature. This is also consistent with observations made in [29]. The preceding discussion and the observations made from Figure 8 and Figure 9 show good sensitivity of the numerical models developed here in VUMAT and VUHARD subroutines to the critical parameters that dictate the shape of the flow stress curve in the DMLS Ti6Al4V (ELI) alloy. The close coincidence of the curves for the numerical models with those for the analytical models is also noted. This creates confidence in the use of these models in the simulation of flow stresses for sample types C, D, and E at various imposed velocities.

**Figure 10:**
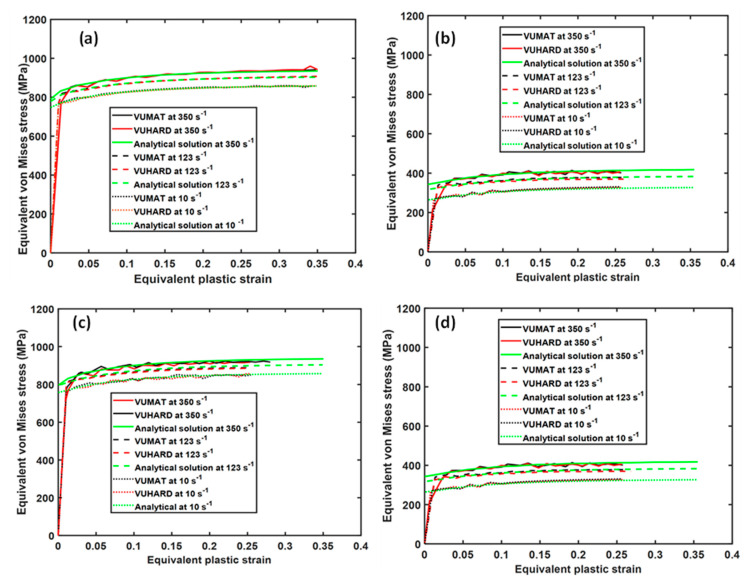
Comparison between the analytical and numerical solutions in VUMAT and VUHARD at three different test velocities and temperatures of (**a,c**) 298 K and (**b**,**d**) 773 K for an 8-node linear brick element in (**a,b**) compression and (**c**,**d**) tension for samples D.

**Figure 11:**
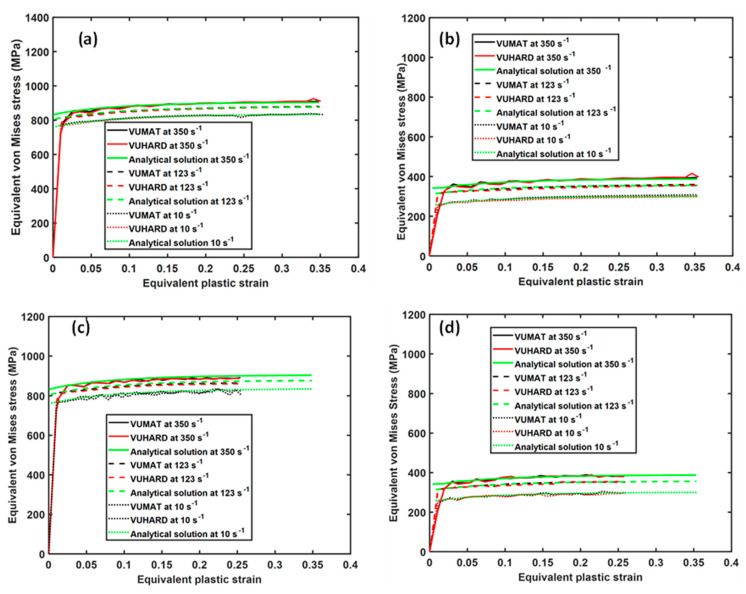
Comparison between the analytical and numerical solutions in VUMAT and VUHARD at three different test velocities and temperatures of (**a**,**c**) 298 K and (**b**,**d**) 773 K for an 8-node linear brick element in (**a**,**b**) compression and (**c**,**d**) tension for samples E.

The values obtained from numerical simulation here were plotted and compared with those obtained from the analytical solutions based on Equation (1) at the same test conditions. The collective results for samples of type C, D, and E eight-node linear brick elements are shown in Figure 9, Figure 10 and Figure 11.

As seen in these three figures, the scaling down and upturn of flow stress at high temperature and with an increase in strain rate, respectively, is well articulated. For the two cases of strain rate and temperature, the results from the numerical models based on the VUMAT and VUHARD subroutines and those derived from the analytical solution developed here are nearly overlapping over most of the equivalent plastic strain range. This confirms that the two subroutines work well in the loading conditions applied.

Both the compression and tensile test simulations showed the same values of flow stress at all strain rates and temperatures, which is anticipated considering the criterion for isotropic yielding used to develop and implement the constitutive models. Even though this was the case, for the same simulation time, there was a significant difference in the maximum equivalent plastic strains attained in the two load conditions, with values of about 0.35 in compression and about 0.25 in tension. The elastic moduli in tension and compression for metals and alloys are normally taken to be the same. Additionally, failure due to the formation of adiabatic shear bands (ASBs) occurs in both high-strain-rate compression and tension. Failure due to monotonic tensile stress occurs first through the formation of pores and cracks and their horizontal spread in the initial brittle fracture and, thereafter, necking before final tearing along the ASBs. Compressive stresses, on the other hand, cause materials to reduce in length and increase in cross section, and failure in most cases is because of the initiation of ASBs. These distinct deformation mechanisms in compression and tension suggest different values of plastic strain for the same applied load, as seen in Figure 9 and Figure 11.

The necking failure mechanism in tension can be studied better using multiple elements, since multiple element models consist of several integration points, and therefore results from various sections of the model can be obtained. A specific geometry of the model can also be used to allow early necking.

### 5.2. Multiple Elements Tests

**Figure 12:**
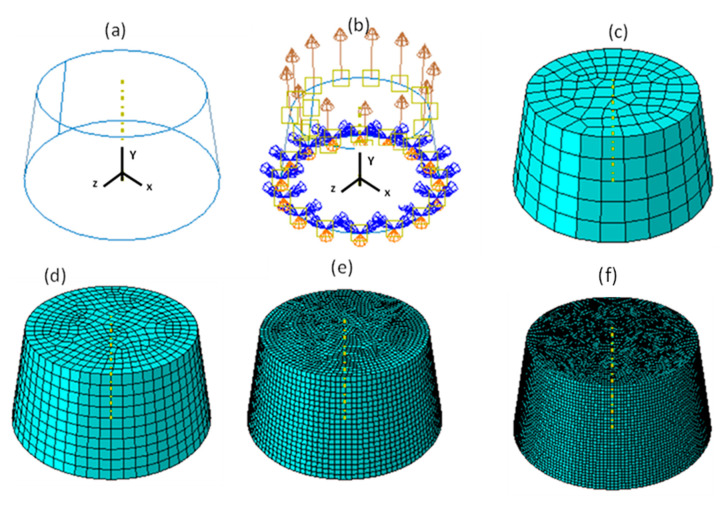
(**a**) A three-dimensional (3D) numerical model for the necking of a circular bar with (**b**) imposed loading and boundary conditions, (**c**) 2 mm mesh size (475 elements), (**d**) 1 mm mesh size (3550 elements), (**e**) 0.5 mm mesh size (28320 elements), and (**f**) 0.25 mm mesh size (126038 elements).

The necking of a circular bar test is an example commonly used in finite element analysis to investigate the necking and softening of a tensile bar [31]. The test was used in the present research to investigate the performance of the VUHARD and VUMAT subroutines developed here under the conditions of necking in tensile loading. The numerical model used was that of the asymmetrical cylinder shown in Figure 12a, with large and small diameters (on the fixed end and at the surface of application of load) of 10 mm and 8 mm, respectively, and a height of 10 mm. The roller boundary condition with y-axis rotational symmetry was applied on the side of the model with a large diameter to constrain the displacement only in the y-direction and to allow rotation only about the y-axis. Tensile loading of the cylinder was realised through imposed instantaneous velocities of 0.1 m/s, 1 m/s, and 4 m/s along the y-axis (the axial direction of the cylinder), shown in Figure 12b, each at a time.

**Figure 13:**
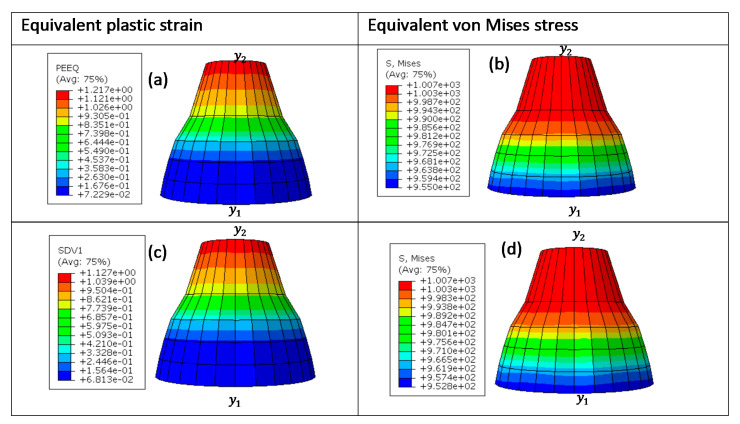
Numerical estimation of (**a**,**c**) equivalent plastic strain and (**b**,**d**) equivalent von Mises stress distribution in (**a**,**b**) VUHARD and (**c**,**d**) VUMAT subroutines developed here, using 2 mm mesh size, at a test velocity of 4 m/s and temperature of 298 K.

**Figure 14:**
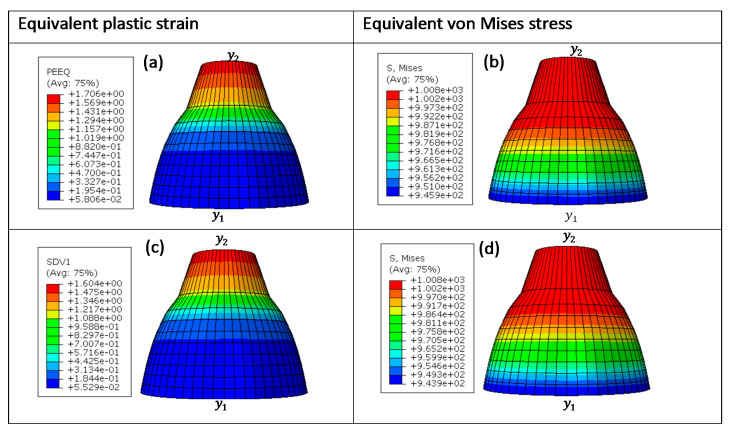
Numerical estimation of (**a**,**c**) equivalent plastic strain and (**b**,**d**) equivalent von Mises stress distribution in (**a**,**b**) VUHARD and (**c**,**d**) VUMAT subroutines developed here, using 1 mm mesh size, at a test velocity of 4 m/s and temperature of 298 K.

**Figure 15:**
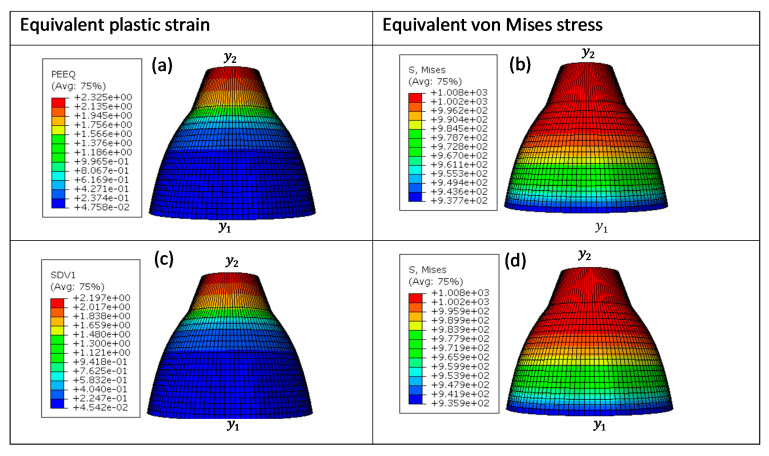
Numerical estimation of (**a**,**c**) equivalent plastic strain and (**b**,**d**) equivalent von Mises stress distribution in (**a**,**b**) VUHARD and (**c**,**d**) VUMAT subroutines developed here, using 0.5 mm mesh size, at a test velocity of 4 m/s and temperature of 298 K.

**Figure 16:**
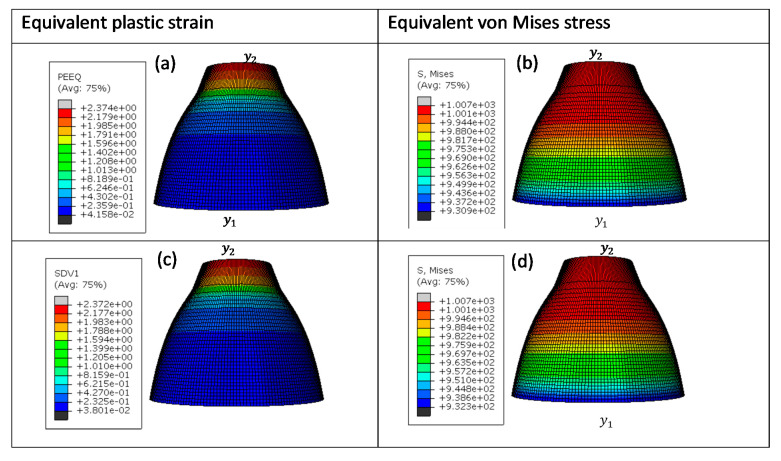
Numerical estimation of (**a**,**c**) equivalent plastic strain and (**b**,**d**) equivalent von Mises stress distribution in (**a**,**b**) VUHARD and (**c**,**d**) VUMAT subroutines developed here, using 0.25 mm mesh size, at a test velocity of 4 m/s and temperature of 298 K.

These four different sizes of meshes yielded 475, 3550, 28,320, and 126,038 linear hexahedral elements of C3D8R type, in order from the largest mesh size to the smallest. Figure 13, Figure 14, Figure 15 and Figure 16 show the distribution of equivalent von Mises stresses and plastic strains obtained using the VUHARD and VUMAT subroutines developed here, for the four different mesh sizes.

A summary of computation times for simulation of the necking bar in this set of simulations is shown in Table 4. The VUMAT subroutine is seen in this table to have more computation time and increments in comparison to VUHARD. The difference in computation time and increments between these two subroutines is possibly due to that fact that they use different integration schemes (discussed later in this section) to update the state variables that could influence the stable time increment. The computation time is also seen to increase with mesh density. For all four sizes of mesh, the VUHARD and VUMAT subroutines display similar distributions of equivalent plastic strain ($\bar{\varepsilon_{p}}$) and von Mises stress ($\bar{\sigma_{p}}$).

From the legends in (a) and (c) of Figure 13 to Figure 16, all four numerical models are seen to experience plastic deformation. The maximum values of equivalent plastic strain and equivalent von Mises stress are located at the tip labelled $y_{2}$ and decline gradually towards $y_{1}$ where they are minimum, as seen in the contours in these figures.

It should be noted that the shape of the undeformed model shown in Figure 12 is that of a truncated cone with the cross-section areas that decrease linearly towards the top surface. Thus, for a given load that is applied at the top surface of the model, the values of equivalent von Mises stress and strain will decline as the cross section increases towards the bottom surface of the original cone.

**Figure 17:**
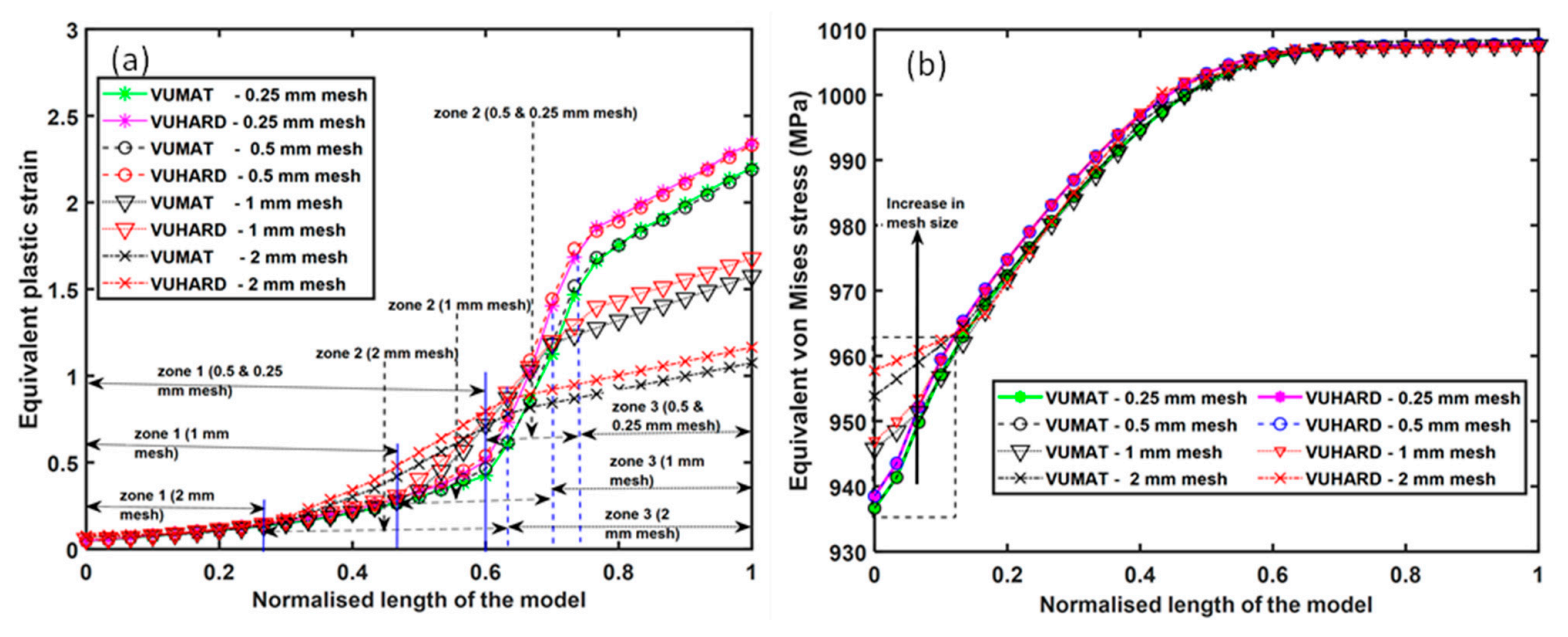
A typical equivalent (**a**) plastic strain and (**b**) von Mises stress distribution along the length of the numerical model with different mesh densities simulated at a velocity of 4 m/s for 0.001 s and at a temperature of 298 K.

**Figure 18:**
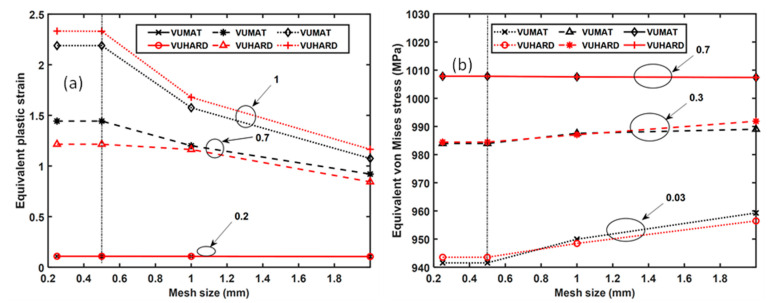
The mesh convergence analysis for equivalent (**a**) plastic strain at model normalised lengths of 0.2, 0.7, and 1 and (**b**) von Mises stress at normalised lengths of the models of 0.03, 0.3, and 0.7 for the VUMAT and VUHARD subroutines.

To better understand the distribution of these two variables for different mesh densities, their values were obtained from elements along the surface from $y_{1}$ to $y_{2}$ at the end of each simulation. These values were then plotted against the normalised length of the deformed model. The resulting curves for different mesh densities are shown in Figure 17, while the results of mesh convergence studies on various sections of the model are presented in Figure 18.

Both the equivalent plastic strains ($\bar{\varepsilon_{p}}$) and von Mises stresses ($\bar{\sigma_{p}}$) show distinct profiles for the three different mesh densities of the model in Figure 17a,b. The $\bar{\varepsilon_{p}}$ profiles exhibit three typical zones, as shown in Figure 17a, which differ in range for different mesh sizes. The first zone shows a slow increase in $\bar{\varepsilon_{p}}$ at increasing rates and its ranges from normalised lengths of 0–0.26, 0–0.47, 0–0.60, and 0–0.60 for models with 2 mm, 1 mm, 0.5 mm, and 0.25 mm mesh sizes, respectively. The slow increase of equivalent strain in this zone is attributed to the large cross-section areas of the model and, therefore, low equivalent von Mises stress acting on elements in the region. The second zone shows a higher increase in $\bar{\varepsilon_{p}}$ with points of inflexion. It ranges from normalised lengths of 0.26 –0.63, 0.47–0.70, 0.60–0.73, and 0.60–0.73 in order of decreasing mesh size. This is the zone that precedes necking. A rapid decrease in the cross-sectional area around this zone resulted in a rapid increase in equivalent von Mises stress and strain. This stage forms the shoulder of the “neck”. The third zone is the necking section of the model and shows an increase in $\bar{\varepsilon_{p}}$ at constant rates, and it ranges from normalised lengths of 0.47–1, 0.70–1, 0.73–1, and 0.73–1 in order of increasing mesh density.

The mesh convergence analysis shown in Figure 18a for the values of equivalent plastic strain obtained at various sections of the model gives rise to several observations. There is convergence of the values of equivalent plastic strain as the mesh density increases in the second and third zones. Lower and higher values of equivalent plastic strain from the VUHARD subroutine in the second and third zones, respectively, are also noted. There is insignificant variation of equivalent plastic strain as the mesh density increases in the first zone, and the results from the VUHARD and VUMAT subroutines are perfectly superimposed in this zone.

From Figure 17b, the influence of mesh density on the distribution of equivalent von Mises stresses is seen to be significant between the normalised lengths of 0 and 0.14. The values of equivalent von Mises stresses are seen to increase with increasing mesh size in this range for both the results obtained using the VUMAT and VUHARD subroutines. However, with 0.5 mm and 0.25 mm mesh sizes, the results from the two subroutines overlap. In the zone between normalised lengths of 0.14 and 0.57, there is a small spread of results obtained for different mesh densities. The variation between the values of equivalent von Mises stresses obtained with coarse and refined mesh models in this region is less than 0.1% of the former. Similar to the preceding zone, the 0.5 mm and 0.25 mm mesh sizes in this zone give results that are indistinguishable. The results obtained in the final zone for normalised lengths of 0.57 to 1 are seen in Figure 17b to be superimposed on top of one another. It is important to note here that for large values of equivalent plastic strain, the flow stress given by the constitutive model developed in this study saturates (no strain hardening), which elucidates the convergence of all these curves in the final zone. This is clearly shown in Figure 18b at a normalised length of the model of 0.7.

The values of equivalent von Mises stresses obtained from the VUHARD subroutine are higher and lower than those from the VUMAT subroutine in the first and second zones at lower mesh densities, respectively. The reverse is true for higher mesh densities, with cross-over points at mesh densities of about 0.85 and 1.2 for the two zones, respectively, while in the last zone, the results from the two subroutines are indistinguishable. Although the results from these two subroutines are very close to one another, the small differences seen in the preceding two figures could be a result of the different integration schemes employed by these two subroutines. The VUHARD subroutine uses an explicit central difference time integration rule built into ABAQUS to generate plastic strain at Gauss points [32], while the VUMAT subroutine uses the radial return method, which belongs to backward or forward integration algorithm methods, in which equivalent plastic strains are evaluated at Gauss points. The difference in their time integration schemes suggests variations in the results obtained due to inherent integration errors associated with each integration scheme. It is important to note that the Euler and central difference methods of solving differential equations are more accurate if the step time is small. The error in the forward and backward integration algorithms is proportional to the size of the time step, while for the central difference method, the error is equal to the square of the size of the time step. Thus, for small time steps (<1 s), the central difference time integration scheme is more accurate than the forward and backward integration methods.

**Figure 19:**
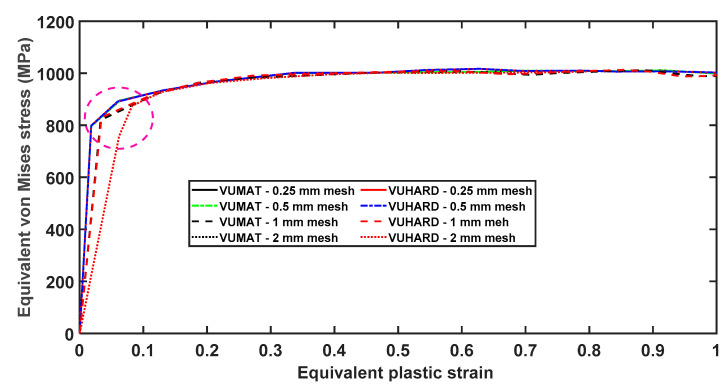
The numerical equivalent von Mises stresses against equivalent plastic strains at a temperature of 298 K and imposed velocity of 4 m/s for different mesh densities obtained from the VUMAT and VUHARD subroutines developed here.

The plots of equivalent plastic strains against equivalent von Mises stresses obtained from the two subroutines are shown in Figure 19 for four different mesh sizes. As seen in this figure, the curves are nearly overlapping for the better part of the flow stress curves. The significant effects of mesh size are revealed at the initial values of equivalent plastic strain (circled in Figure 19), with the 2 mm mesh size giving a much higher value of about 0.083 equivalent plastic strain in comparison to the 0.25 mesh size, which has a value of about 0.02. However, the curves for 0.25 mm and 0.5 mm mesh sizes are indistinguishable at all values of strain, which is consistent with the observations made from Figure 17 and Figure 18.

It is not surprising to see the flow stress saturating at the equivalent plastic strain of 0.4 and onward for the VUMAT and VUHARD subroutines, since the implemented model was not developed to take care of unloading conditions or even evolution of damage. During the initial stage of plastic deformation, materials experience work-hardening where flow stress increases with increasing strain. Work-hardening normally occurs due to generation and accumulation of dislocations with increasing plastic strain. However, as previously reported in the authors’ previous work in [28], materials loaded at a high strain rate experience “thermal softening”. Thermal softening causes dynamic recovery where dislocations begin to annihilate. If the rate of annihilation of dislocations equals the rate of generation of dislocations by strain hardening, then the flow stress saturates. However, if the rate of annihilation of dislocations exceeds the rate at which dislocations are generated, materials experience decreasing flow stress with increasing plastic strain, a phenomenon commonly referred to as “strain softening”. The threshold value of the accumulation of dislocations for the three forms of DMLS Ti6Al4V (ELI) samples was calibrated as 8.3 × 10^15^ m^−2^ in [29]. The formation of ASBs is the precursor to material failure due to the formation and growth of microvoids and cracks along them [28]. The failure processes at high strain rates that lead to instabilities can be reflected in the numerical models through extension of the analytical model to predict the evolution of such damage. However, this was not within the scope of the present research, and future research is expected to focus on integrating the evolution of damage into the constitutive numerical model developed here.

The foregoing graphs and related discussions suggest that mesh density does influence the strain and stress distribution in a numerical model. However, this does not appear to significantly affect the VUMAT and VUHARD subroutines differently. This is evident from the fact that for the four different mesh densities used here, the VUMAT and VUHARD subroutines’ equivalent von Mises stress–strain curves are nearly superimposed on top of one another for most of the flow stress curves. Furthermore, the flow stress curves and the distribution of equivalent strain and von Mises stress given by the models with 0.25 mm and 0.5 mm mesh sizes are indistinguishable. The values of equivalent plastic strain and von Mises stress at various sections of the model were also noted to correspond for these two mesh sizes. Given that more computation resources (space and time) are required as mesh density increases, the mesh of size of 0.5 mm was selected for simulation of the necking of a cylindrical numerical model at different strain rates and temperatures of 298 K and 773 K.

**Figure 20:**
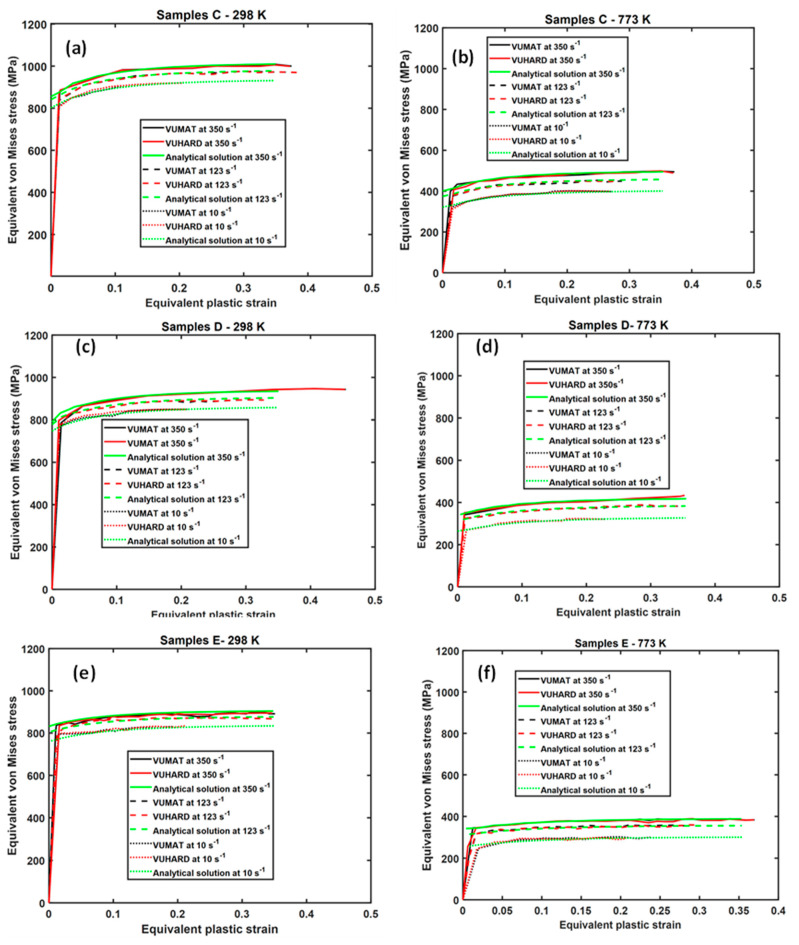
Comparison between the analytical and numerical solutions using the VUMAT and VUHARD subroutines at three test velocities and a temperature of (**a**,**c**,**e**) 298 K and (**b**,**d**,**f**) 773 K for a multiple element cylindrical model of (**a**,**b**) samples C, (**c**,**d**) samples D, and (**e**,**f**) samples E.

The results obtained from the last elements near the point $y_{2}$ (shown in Figure 16) were compared with the analytical solutions obtained from Equation (1) at different strain rates. The flow stress curves obtained from numerical simulation and the analytical constitutive model for sample types C, D, and E, in this case, are summarized in Figure 20.

Similar to the result from the single element test reported earlier on, the curves for the analytical solution and those from the VUMAT and VUHARD subroutines are nearly superimposed on top of one another at temperatures of 298 K and 773 K and respective strain rates, as seen in Figure 20. That the curves in this figure are nearly overlapping indicates that both subroutines are accurate in modelling a multiple element cylinder.

The foregoing discussion suggests that both subroutines implemented in this study can reliably be used to simulate the mechanical properties of various microstructures of additively manufactured Ti6Al4V parts. However, it has also been noted that the VUMAT subroutine is slower and requires more computational resources compared to the VUHARD subroutine. This is a clear indication that the different integration schemes employed by these subroutines influence the stable time increment.

## 6. Conclusions

The implementation and verification processes of the VUMAT and VUHARD subroutines for numerical simulations of plastic deformation of various forms of DMLS Ti6Al4V (ELI) were presented and discussed in this article. The following conclusions are deduced from the research:

- Using single element numerical models and at a test velocity of 4 m/s, both VUMAT and VUHARD subroutines showed fairly similar values of average plastic strain rate of approximately 350 s^−1^.
- The equivalent plastic strain and von Mises stress generated from the two subroutines for the single element numerical model also coincide for a large part of the simulation time.
- The single element numerical models developed in this study and simulated using the developed VUMAT and VUHARD subroutines were shown to be sensitive to the critical model parameters of initial dislocation density ($\rho_{o}$), test temperature (T), and strain hardening and the dynamic recovery parameters h and $k_{2}$, respectively.
- The results of simulation of samples C, D, and E using single element numerical models based on the VUMAT and VUHARD subroutines and those derived from the analytical solutions at the same conditions of strain rate and temperature were nearly overlapping over most of plastic strain range.
- For the same simulation time, the single element numerical models showed a significant difference in the maximum equivalent plastic strain attained in the two load conditions of compression and tension, with values of about 0.35 in compression and about 0.25 in tension.
- For multiple element models with different mesh sizes, both the VUMAT and VUHARD subroutines displayed similar distributions of equivalent plastic strain and von Mises stress.
- The equivalent plastic strain and von Mises stress profiles for multiple element numerical models exhibited three typical zones which varied in range for different mesh sizes.
- The values of equivalent von Mises stress obtained in VUHARD and VUMAT subroutines varied in the first and second zones for different mesh sizes. The small variations seen in these results were attributed to the different integration schemes employed by these subroutines.
- The results obtained from both subroutines for the models with 0.25 mm and 0.5 mm mesh sizes were indistinguishable.
- Comparisons between the results obtained for the multiple element numerical modelling of samples C, D, and E and the curves obtained from analytical solutions at the same conditions of temperature and strain rate were nearly superimposed.

It is recommended that future research aims at carrying out complex dynamic simulations of experimental tests, such as split-Hopkinson bar pressure tests, the Taylor impact test, and blast loading of plates using the developed VUMAT and VUHARD subroutines. The microstructure-sensitive model implemented as a material subroutine in this study should be extended to include some form of failure criterion or damage model.

## Figures and Tables

**Table 1:** Numerical model input parameters obtained from the DMLS Ti6Al4V (ELI) microstructures.

Sample Type	Average Grain Size (μm)	Average Initial Dislocation Density $\left(m^{-2}\right)$
C	2.5	5.73 × 1014
D	6.0	5.09 × 1014
E	9.0	7.00 × 1014

**Table 2:** The prescribed and fitting parameters of the model.

Prescribed Parameters	Value and Units	Fitted Parameters	Values and Units
Boltzmann constant $(k_{b}$)	1.38 × 10^−23^ m^2^kgs^−2^ k^−1^	$\sigma^{o}$	1063.2 MPa
Shear modulus (μ)	$49.02-5.821/\left(e^{\frac{181}{T}}-1\right)\;\left(GPa\right)$	$g_{0i}$	0.25
Burgers vector (b)	$2.95\times 10^{-10}\;m$	h	8.3 × 10^15^ m^−2^
Reference strain rate $\left({\dot{\varepsilon }}_{o}\right)$	10^7^ s^−1^	$k_{2}$	10
Hall–Petch constant ($K_{H-P}$)	$0.328\;\mathrm{MPa}\cdot m^{1/2}$	*ζ*	(MPa)
P	1	Samples C	207
Q	2	Samples D	210
Taylor factor (M)	3	Samples E	210
α	0.2	χ	
		Samples C	0.00032
		Samples D	0.00030
		Samples E	0.00020

**Table 3:** The VUHARD and VUMAT subroutine and the ABAQUS material property definitions.

Variables	VUHARD	ABAQUS	VUMAT	ABAQUS
Elastic modulus	-	-	e	Props (1)
Poisson’s ratio	-	-	xnu	Props (2)
K/b^3^	Kb3	Props (1)	Kb3	Props (3)
goi	Goi	Props (2)	goi	Props (4)
${\dot{\varepsilon }}_{o}$	ddeqps0	Props (3)	ddeqps0	Props (5)
$\sigma^{o}$	Theta	Props (4)	theta	Props (6)
p	P	Props (5)	p	Props (7)
q	Q	Props (6)	q	Props (8)
$\rho_{o}$	e0	Props (7)	e0	Props (9)
h	H	Props (8)	h	Props (10)
k2	k2	Props (9)	k2	Props (11)
α × b × M	Abm	Props (10)	abm	Props (12)
$K_{H-p}$	Khp	Props (11)	Khp	Props (13)
$\alpha_{1}$	Alpha	Props (12)	alpha	Props (14)
ζ	Ala	Props (13)	ala	Props (15)
t	T	Props (14)	t	Props (16)
U_0_	U0	Props (15)	U0	Props (17)
D	D	Props (16)	D	Props (18)
T_0_	T0	Props (17)	T0	Props (19)

**Table 4:** Computation time and number of increments for different mesh densities.

Subroutine	VUHARD		VUMAT	
Mesh Size (mm)	Time (s)	Increments	Time (s)	Increments
2	6.3	92	9.2	96
1	59.3	200	63.2	219
0.5	177.2	447	396.6	629
0.25	1676	900	1876	1037

**Figure A1:**
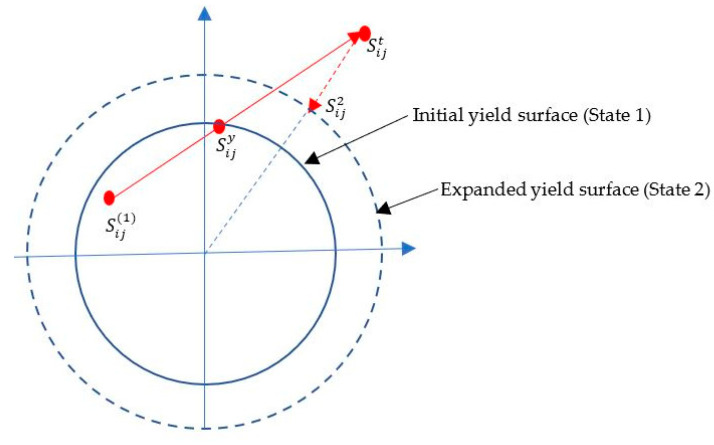
The radial return method for isotropic/von Mises hardening.

## Supplementary Materials

The following are available online at https://www.mdpi.com/article/10.3390/ma15010372/s1, S1: The developed code for VUHARD subroutine, S2: The developed code for VUMAT subroutine.

## Appendix A. The von Mises Yield Criterion (*J_2_*) and Radial Return Algorithm

### Appendix A.1. J_2_ Plasticity

A stress tensor ($\sigma_{ij}$) consisting of nine components is used in continuum mechanics to define the state of stress at a point/element inside a material under application of load [33,34]. The mean stress ($\sigma_{m}$), which is also known as hydrostatic or equivalent stress, is expressed as

(A1) $$\sigma_{m}=\frac{1}{3}\;\left(\sigma_{xx}+\sigma_{yy}+\sigma_{zz}\right)$$

where $\sigma_{xx}$, $\sigma_{yy}$, and $\sigma_{zz}$ are principal stresses. The deviatoric stress tensor ($S_{ij}$) is given by the difference between the stress tensor ($\sigma_{ij}$) and the hydrostatic stress ($\sigma_{m}\delta_{ij}$) expressed as

(A2) $$S_{ij}=\sigma_{ij}-\sigma_{m}\delta_{ij}$$

In a similar manner to the deviatoric stress tensor, the deviatoric strain tensor ($e_{ij}$) is given by the difference between the strain tensor ($\varepsilon_{ij}$) and the mean strain ($\varepsilon_{m}\delta_{ij}$):

(A3) $$e_{ij}=\varepsilon_{ij}-\frac{1}{3}\varepsilon_{kk}\delta_{ij}=\varepsilon_{ij}-\varepsilon_{m}\delta_{ij}$$

where $\varepsilon_{m}=\frac{1}{3}\left(\varepsilon_{xx}+\varepsilon_{yy}+\varepsilon_{xx}\right)$ is the mean strain, and $\varepsilon_{kk}$ is the volumetric strain or dilatation. The elastic stress tensor for homogenous and isotropic materials is defined by two Lamé constants which are material dependent quantities denoted by the symbols λ (lambda) and μ (shear modulus) that arise in stress–strain relationships [35]. Using these two Lamé constants, the stress tensor can be expressed as

(A4) $$\sigma_{ij}=2\mu \varepsilon_{ij}+\lambda \varepsilon_{kk}\delta_{ij}$$

The rate of deformation can be described as the sum of the elastic (${\dot{\varepsilon }}_{ij}^{e}$) and plastic (${\dot{\varepsilon }}_{ij}^{p}$) strain rate components given as

(A5) $${\dot{\varepsilon }}_{ij}={\dot{\varepsilon }}_{ij}^{e}+{\dot{\varepsilon }}_{ij}^{p}$$

Any plasticity numerical model must distinguish between the elastic and plastic responses. Therefore, some formulations are required to define the (a) yield surface, where yielding occurs; (b) flow rule, which defines the direction of the plastic deformation; and (c) hardening law, which describes the variation of flow with plastic deformation [27]. Thus, the yield surface is defined by a function of the form

(A6) $$f\left(\sigma_{ij},{\dot{\varepsilon }}_{ij}^{p},\;T,\;k^{n}\;\right)=0$$

The yield surface is thus a function of the stress tensor ($\sigma_{ij}$)*,* plastic strain state (${\dot{\varepsilon }}_{ij}^{p}$), temperature (T), and hardening parameter$\;k^{n}$. The stage for which f≤0 indicates that the material is within the elastic region. On the other hand, the condition f>0 exists in the case of rate-dependent plasticity, resulting in stress states that lie beyond the yield surface [27,36]. For the isotropic von Mises yield criterion, the yield surface can be expressed in terms of the second deviatoric stress invariant *J_2_*, and thus the yield function can be written as

(A7) $$f\left(\;J_{2}\right)=0$$

From the von Mises failure criterion (distortion energy theory) [34,35] and the second deviatoric stress invariant ***J_2_***, Equation (A7) can be written as

(A8) $$\sigma_{y}-\sqrt{3J_{2}}=0\;\mathrm{or}\;\sigma_{y}-\sqrt{\frac{3}{2}S_{ij}S_{ji}}=0$$

where $\sigma_{y}$ is the yield stress or the value of stress of the last increment. This scalar quantity of stress obtained from the deviatoric stress is commonly referred to as the von Mises equivalent stress, q, computed in most finite element analysis packages such as ABAQUS *viz* [27]:

(A9) $$q=\sqrt{3/2\left(S_{ij}S_{ji}\right)}$$

Likewise, the scalar quantity of strain from the deviatoric strain, commonly referred to as equivalent strain, is expressed as [27]

(A10) $$\varepsilon_{eq}=\sqrt{2/3\left(e_{ij}e_{ij}\right)}$$

When $\sigma_{y}-\sqrt{q}>0$, the state of the material is no longer elastic, and therefore a plastic correction must be performed. In other words, the equivalent increment in inelastic strain must be calculated to return the isotropic stress state to a von Mises yield surface. The radial return mapping algorithm introduced by Wilkins [37] is the common method that uses the von Mises yield surface to define the increment of plastic strain necessary to return the stress state to the yield surface after an increment of trial stress ($S_{ij}^{t}$).

### Appendix A.2. The Radial Return Algorithms

It is a very rare situation for most materials to behave perfectly plastic, the state of which makes the yield surface expand during plastic deformation. In radial return mapping, the stress is updated with an assumption that the response is elastic, and if it is beyond the yield surface, the stress is projected onto the closest point on the yield surface. In elastoplastic deformation, the total change in strain (Δε) can be expressed as the sum of the elastic ($\Delta \varepsilon^{e}$) and plastic strain ($\Delta \varepsilon^{p}$) changes:

(A11) $$\Delta \varepsilon =\Delta \varepsilon^{e}+\Delta \varepsilon^{p}$$

If the change in strain tensor ($\Delta \varepsilon_{ij}$) has been derived for the Gauss integration point from the current iteration, then the trial stress $S_{ij}^{t}$ can be obtained as

(A12) $$S_{ij}^{t}=S_{ij}^{\left(1\right)}+2\mu \Delta \varepsilon_{ij}$$

where $S_{ij}^{\left(1\right)}$ is the old trial stress obtained from previous iteration, and μ is the shear modulus of the material. The actual stress due to plastic deformation, obtained at the end of the increment, is not the trial stress but rather the one returned to the yield surface, as seen in Figure A1.

From Figure A1, the actual deviatoric stress can be expressed as

(A13) $$S_{ij}^{\left(2\right)}=S_{ij}^{\left(1\right)}+2\mu \Delta \varepsilon_{ij}^{e}=S_{ij}^{\left(1\right)}+2\mu \left(\Delta \varepsilon_{kl}-\Delta \varepsilon_{kl}^{p}\right)=S_{ij}^{t}-2\mu \Delta \varepsilon_{kl}^{p}$$

The correction, also referred to as return stress ($S_{ij}^{r}$), is expressed as

(A14) $$S_{ij}^{r}=2\mu \Delta \varepsilon_{kl}^{p}$$

The return stress ($S_{ij}^{r}$) leads to the name “radial return” method. The implicit integration scheme [34] is presented in Appendix B.

## Appendix B. The Implicit Integration Scheme to Compute for Equivalent Plastic Strain

The deviatoric stress during plastic deformation can be expressed as [27]

(A15) $$S_{ij}^{\left(2\right)}=S_{ij}^{\left(1\right)}+2\mu \Delta \varepsilon_{ij}^{e}=S_{ij}^{\left(1\right)}+2\mu \left(\Delta \varepsilon_{kl}-\Delta \varepsilon_{kl}^{p}\right)=S_{ij}^{t}-2\mu \Delta \varepsilon_{kl}^{p}$$

where $S_{ij}^{\left(2\right)}$ and $S_{ij}^{\left(1\right)}$ are the new and old deviatoric stress; $\Delta \varepsilon_{ij}^{e}$, $\Delta \varepsilon_{kl}$, and $\Delta \varepsilon_{kl}^{p}$ are the increment in deviatoric elastic strain, equivalent strain, and equivalent plastic strain, respectively; $S_{ij}^{t}$ is the trial stress; and μ is the shear modulus. The return stress from Equation (A15) is

(A16) $$S_{ij}^{r}=2\mu \Delta \varepsilon_{kl}^{p}$$

To evaluate the value of the return stress $S_{ij}^{r}$, and therefore the deviatoric stress $S_{ij}^{\left(2\right)}$, the increment of the plastic component $\Delta \varepsilon_{kl}^{p}$, and therefore the increment in the equivalent plastic strain $\Delta \epsilon_{e}^{p}$, must be determined. Thus,

(A17) $$\Delta \varepsilon_{kl}^{p}=\left(\frac{\partial f}{\partial \sigma_{kl}}\right)\partial \epsilon^{p}=\frac{3}{2}\frac{S_{ij}^{\left(2\right)}}{q^{\left(2\right)}}\Delta \epsilon_{e}^{p}$$

where $\partial \epsilon^{p}$ is the equivalent plastic strain increment, and $q^{\left(2\right)}$ is the new equivalent von Mises stress. Equations (A15) and (A17) can be combined into

(A18) $$S_{ij}^{\left(2\right)}=S_{ij}^{t}-2\mu \frac{3}{2}\frac{S_{ij}^{\left(2\right)}}{q^{\left(2\right)}}\Delta \epsilon_{e}^{p}=S_{ij}^{t}-3\mu \frac{S_{ij}^{\left(2\right)}}{q^{\left(2\right)}}\Delta \epsilon_{e}^{p}$$

Thus, the new deviatoric stress is given by

(A19) $$S_{ij}^{\left(2\right)}=\frac{S_{ij}^{t}}{1+3\mu \frac{\partial \epsilon^{p}}{q^{\left(2\right)}}}$$

The new equivalent von Mises stress can be obtained from Equation (A19), thus making Equation (A8) of the appendix

(A20) $$q^{\left(2\right)}=\sqrt{\frac{3}{2}S_{ij}^{2}S_{ij}^{2}}=\frac{\sqrt{\frac{3}{2}S_{ij}^{t}S_{ij}^{t}}}{1+3\mu \frac{\Delta \epsilon_{e}^{p}}{q^{\left(2\right)}}}=\frac{\sigma_{e}^{t}}{1+3\mu \frac{\Delta \epsilon_{e}^{p}}{q^{\left(2\right)}}}$$

Since, at the yielding surface, $q^{\left(2\right)}=\sigma_{y}^{\left(2\right)}$ (where$\;\sigma_{y}^{\left(2\right)}$ is the new stress computed from the constitutive equation),

(A21) $$\sigma_{y}^{\left(2\right)}=\sigma_{e}^{t}-3\mu \Delta \epsilon_{e}^{p}\;\mathrm{or}\;\sigma_{e}^{t}-3\mu \Delta \epsilon_{e}^{p}-\sigma_{y}^{\left(2\right)}=0$$

Equation (A21) is a non-linear yield function, and the increment of plastic strain $\partial \epsilon^{p}$ between states 1 (old state) and 2 (new state) must be computed. The iteration methods of finding solutions of non-linear functions can be used to do this. Alternatively, a non-iteration method can be used to solve for $\partial \epsilon^{p}$ [27] if the plastic flow stress occurring between the old and new state can be considered. The flow stress at the new state can be expressed explicitly using the flow stress at the old state as

(A22) $$\sigma_{y}^{\left(2\right)}=\sigma_{y}^{\left(1\right)}+H\Delta \epsilon_{e}^{p}$$

where H denotes the rate of change in flow stress with plastic strain. Equations (A21) and (A22) can be combined to solve for $\partial \epsilon^{p}$, the new deviatoric stress, and the stress tensor as

(A23) $$\Delta \epsilon_{e}^{p}=\frac{\sigma_{e}^{t}-\sigma_{y}^{\left(1\right)}}{3\mu +H}$$

(A24) $$factor=\frac{\sigma_{y}^{\left(2\right)}}{\sigma_{e}^{t}}=\frac{\sigma_{y}^{\left(2\right)}}{\left(\sigma_{y}^{\left(2\right)}+3\mu \Delta \epsilon_{e}^{p}\right)}\;$$

(A25) $$S_{ij}^{new}=factor\;x\;S_{ij}^{t}$$

(A26) $$\sigma_{ij}^{new}=S_{ij}^{new}+\sigma_{m}$$

Once the increase in equivalent plastic strain is computed, the proportionality factor $\left(\sigma_{y}^{\left(1\right)}/\sigma_{e}^{t}\right)$ is calculated using Equation (A24), and the trial deviatoric stresses are scaled back to the yield surface using Equation (A25). The new stress tensor is then updated by adding the scaled trial deviatoric stresses to the hydrostatic stress in Equation (A26).
