# Supplementary material for: Development of VUMAT and VUHARD Subroutines for Simulating the Dynamic Mechanical Properties of Additively Manufactured Parts
Source: Materials (Basel). 2022 Jan 5;15(1):372. doi: 10.3390/ma15010372 (PMC8746190; doi:10.3390/ma15010372)
Supplement: Supplementary file 1 [file materials-15-00372-s001.zip › materials-1497887-supplementary.pdf]

S1. The developed code for VUHARD subroutine

```
C ********************************************************************
       subroutine vuhard (
C Read only -
     *     nblock,
     *     jElem, kIntPt, kLayer, kSecPt,
     *     lAnneal, stepTime, totalTime, dt, cmname,
     *     nstatev, nfieldv, nprops,
     *     props, tempOld, tempNew, fieldOld, fieldNew,
     *     stateOld,
     *     eqps, eqpsRate,
C Write only -
     *     yield, dyieldDtemp, dyieldDeqps,
     *     stateNew)
C
      include 'vaba_param.inc'
C
           dimension props(nprops), tempOld(nblock), tempNew(nblock),
     1   fieldOld(nblock,nfieldv), fieldNew(nblock,nfieldv),
     2   stateOld(nblock,nstatev), eqps(nblock), eqpsRate(nblock),
     3   yield(nblock), dyieldDtemp(nblock), dyieldDeqps(nblock,2),
     4   stateNew(nblock,nstatev), jElem(nblock)
C
        character*80 cmname
C *************************************************************************
C Print the constitutive model parameters
          kb3     = props(1)
          goi     = props(2)
         ddeqps0 = props(3)
          theta   = props(4)
          p       = props(5)
          q       = props(6)
          e0      = props(7)
          h       = props(8)
          k2      = props(9)
          abm     = props(10)
          Khp     = props(11)
          alpha   = props(12)
          ala     = props(13)
          t       = props(14)
          U0      = props(15)
          D       = props(16)
          T0      = props(17)
C**********************************************************************
C    Print out material properties.
C**********************************************************************
           write (*,*)'The model constant parameters'
           write (*,*)'kb3=',kb3
           write (*,*)'goi=',goi
           write (*,*)'ddeqps0=',ddeqps0
           write (*,*)'theta=',theta
           write (*,*)'p=',p
           write (*,*)'q=',q
           write (*,*)'e0=',e0
```

```
           write (*,*)'h=',h
           write (*,*)'k2=',k2
           write (*,*)'abm=',abm
           write (*,*)'Khp=',Khp
           write (*,*)'alpha=',alpha
           write (*,*)'ala=',ala
           write (*,*)'laths thickness=',t
           write (*,*)'U0=',U0
           write (*,*)'D=',D
           write (*,*)'T0=',T0
C
C The Hall-Petch relationship from the alpha length thickness (t)
C
          Khp_D=Khp/sqrt(t)
          write (*,*)'Khp_D=',Khp_D

          do k = 1, nblock
             epps = eqps(k)
             temp = tempOld(k)
             write(*,*)'temp=',temp
C   Compute for Temperature dependent shear modulus
          if (temp.eq.0.D0) then
           U=U0
          else
           U=U0-((D/(exp(T0/temp)-1.0))) *10**3
         end if
           write (*,*),'U=',U
C
C   Compute for the thermal part of flow stress
C
        thermal1=1-((kb3*temp)/(goi*U)*log(ddeqps0/ddeqps))**(1.0/q)
         thermal2=theta*thermal1**(1.0/p) *U/U0
          write (*,*)'thermal1=',thermal1
           write (*,*)'thermal2=',thermal2
C

C   Compute for athermal part of flow stress
C
         athermal1=(1-exp(-k2*epps))*h/k2
           athermal2=e0*exp(-k2*epps)
             athermal3=ala*(1-exp(-alpha*ddeqps))
         athermal4=sqrt(athermal1+athermal2) *abm*U +athermal3 + Khp_D
          write (*,*)'athermal1=',athermal1
           write (*,*)'athermal2=',athermal2
            write (*,*)'athermal3=',athermal3
             write (*,*)'athermal4=',athermal4
C
C   Compute for Total flow stress at different level of epps
C
         flowstress=thermal2+athermal4
          write (*,*)'flowstress=',flowstress
C
C   Compute for Partial derivative of Total flowstress w.r.t epps
C
          dathermal1=abm*U/2*(athermal1+athermal2)**(-1.0/2)
           dathermal2=h*exp(-k2*epps)-(e0*k2*exp(-k2*epps))
          dathermal3=dathermal1*dathermal2
```

```
            write (*,*)'dathermal1=',dathermal1
             write (*,*)'dathermal2=',dathermal2
              write (*,*)'dathermal3=',dathermal3
C
C Assign an array of Partial derivative of Total flowstress w.r.t epps.
C
                dyieldDeqps(k,1) = dathermal3
                 write(*,*)'dyieldDeqps(k,1)=',dyieldDeqps(k,1)
C
C Compute the Partial derivatives of Total flowstress w.r.t ddeqps.
C
         eqpsRate(k)= ddeqps
         p2=(1.0-p)/p
         q2=(1.0-q)/q
         ddthermal1=(theta*temp*kb3)/(p*q*U*ddeqps)
         ddthermal2=(1-(log(ddeqps0/ddeqps) *kb3*temp/goi/U)**(1.0/q)) **p2
           ddthermal3=(log(ddeqps0/ddeqps) *kb3*temp/goi/U) **q2
            ddthermal4= alpha*exp(-alpha*ddeqps)
         ddthermal5=ddthermal1*ddthermal2*ddthermal3+ddthermal4
         write (*,*)'ddthermal1=',ddthermal1
          write (*,*)'ddthermal2=',ddthermal2
           write (*,*)'ddthermal3=',ddthermal3
            write (*,*)'ddthermal4=',ddthermal4

C
C Assign an array for partial derivatives of Total flowstress w.r.t ddeqps.
C
              dyieldDeqps(k,2) = ddthermal5
               write (*,*)' dyieldDeqps(k,2)=', dyieldDeqps(k,2)
C
C Compute and return the yield stress.
C
         yield(k) = flowstress

         write (*,*)' yield(k)=',  yield(k)
         write (*,*)'eqps=',eqps(k)
         write (*,*)'eqpsRate(k)=', eqpsRate(k)
          end do
        return
       end
```

S2. The developed code for VUMAT subroutine

```
       subroutine vumat(
C Read only (unmodifiable)variables -
     1  nblock, ndir, nshr, nstatev, nfieldv, nprops, jInfoArray,
     2  stepTime, totalTime, dtArray, cmname, coordMp, charLength,
     3  props, density, strainInc, relSpinInc,
     4  tempOld, stretchOld, defgradOld, fieldOld,
     5  stressOld, stateOld, enerInternOld, enerInelasOld,
     6  tempNew, stretchNew, defgradNew, fieldNew,
C Write only (modifiable) variables -
     7  stressNew, stateNew, enerInternNew, enerInelasNew )
C
      include 'vaba_param.inc'
      parameter (i_info_AnnealFlag = 1,
     *     i_info_Intpt    = 2, ! Integration station number
     *     i_info_layer  = 3, ! Layer number
     *     i_info_kspt   = 4, ! Section point number in current layer
     *     i_info_effModDefn = 5, ! =1 if Bulk/ShearMod need to be defined
     *    i_info_ElemNumStartLoc   = 6) ! Start loc of user element number
C
      dimension props(nprops), density(nblock), coordMp(nblock,*),
     1  charLength(nblock), dtArray(2*(nblock)+1),
strainInc(nblock,ndir+nshr),
     2  relSpinInc(nblock,nshr), tempOld(nblock),
     3  stretchOld(nblock,ndir+nshr),
     4  defgradOld(nblock,ndir+nshr+nshr),
     5  fieldOld(nblock,nfieldv), stressOld(nblock,ndir+nshr),
     6  stateOld(nblock,nstatev), enerInternOld(nblock),
     7  enerInelasOld(nblock), tempNew(nblock),
     8  stretchNew(nblock,ndir+nshr),
     8  defgradNew(nblock,ndir+nshr+nshr),
     9  fieldNew(nblock,nfieldv),
     1  stressNew(nblock,ndir+nshr), stateNew(nblock,nstatev),
     2  enerInternNew(nblock), enerInelasNew(nblock), jInfoArray(*)
C
      character*80 cmname
C
      pointer (ptrjElemNum, jElemNum)
      dimension jElemNum(nblock)
C
      lAnneal = jInfoArray(i_info_AnnealFlag)
      iLayer = jInfoArray(i_info_layer)
      kspt   = jInfoArray(i_info_kspt)
      intPt  = jInfoArray(i_info_Intpt)
      iUpdateEffMod = jInfoArray(i_info_effModDefn)
      iElemNumStartLoc = jInfoArray(i_info_ElemNumStartLoc)
      ptrjElemNum = loc(jInfoArray(iElemNumStartLoc))
C

C Material property definitions

              e      =  props(1)
              xnu    =  props(2)
              kb3    =  props(3)
              goi    =  props(4)
```

```
              ddeqps0 =  props(5)
               theta  =  props(6)
               p      =  props(7)
               q      =  props(8)
               e0     =  props(9)
               h      =  props(10)
               k2     =  props(11)
               abm    =  props(12)
               Khp    =  props(13)
               alpha  =  props(14)
               ala    =  props(15)
               t      =  props(16)
               U0     =  props(17)
               D      =  props(18)
               T0     =  props(19)

C
C Find the shear and bulk modulus.
C
                μ =e/(two*(one+xnu))
                bulk=e/(three*(one-two*xnu))
                alamda=e*xnu/((1+xnu) *(1-2*xnu))
                write (*,*)'μ=',μ
                write (*,*)'bulk=',bulk
                write (*,*)'alamda=',alamda
C
        if ((stepTime+totalTime).eq.0.0) then
           do i=1,nblock
             trace=strainInc(i,1)+strainInc(i,2)+strainInc(i,3)
              write (*,*)'trace=',trace
C New stress tensor due to elastic behaviour

stressNew(i,1)=stressOld(i,1)+two*G*strainInc(i,1)+alamda*trace

stressNew(i,2)=stressOld(i,2)+two*G*strainInc(i,2)+alamda*trace

stressNew(i,3)=stressOld(i,3)+two*G*strainInc(i,3)+alamda*trace
              stressNew(i,4)=stressOld(i,4)+two*G*strainInc(i,4)
              if (nshr.gt.1) then
              stressNew(i,5)=stressOld(i,5)+two*G*strainInc(i,5)
              stressNew(i,6)=stressOld(i,6)+two*G*strainInc(i,6)
              end if
                    write(*,*)'stressNew(i,1)=',stressNew(i,1)
                    write(*,*)'stressNew(i,2)=',stressNew(i,2)
                    write(*,*)'stressNew(i,4)=',stressNew(i,4)
           end do
      else
            do i=1,nblock
C
C Initialize the yield stress, plastic strain, and strain rate of the last
C   increment.
C
                deqps    = stateOld(i,1)
                ddeqps   = stateOld(i,2)
                yieldOld = stateOld(i,3)
                Temp     = TempOld(i)
C The Hall-Petch relationship from the alpha length thickness (t)
```

```
          Khp_D=Khp/sqrt(t)
          write (*,*)'Khp_D=',Khp_D
C Initialize small initial plastic strain.
                depl=1e-8
C Compute for Temperature dependent shear modulus
          if (temp.eq.0. D0) then
           U=U0
          else
              U=U0-((D/(exp(T0/temp)-1.0))) *10**3
         end if
           write (*,*),'U=',U
C
C Compute for yielding: Athermal and thermal components from the
C   constitutive model.
C
C Thermal part of flow stress

       thermal1=1-((kb3*temp)/(goi*U) *log(ddeqps0/(ddeqps))) **(1.0/q)
        thermal2=theta*thermal1**(1.0/p) *U/U0
           write(*,*)'thermal1=',thermal1
            write(*,*)'thermal2=',thermal2
C                  Athermal part of flow stress
        athermal1=(1-exp(-k2*deqps)) *h/k2
          athermal2=e0*exp(-k2*deqps)
            athermal3=ala*(1-exp(-alpha*(ddeqps)))
             athermal4=sqrt(athermal1+athermal2) *abm*U +athermal3 + Khp_D
         write (*,*)'athermal1=',athermal1
          write (*,*)'athermal2=',athermal2
           write (*,*)'athermal3=',athermal3
            write (*,*)'athermal4=',athermal4
C
C Compute for old yield stress
C
        yieldOld=thermal2+athermal4
         write (*,*)'yieldOld=',yieldOld
C Compute for Partial derivative of yield stress w.r.t equivalent plastic
C strain (hard).
C
         hard1=abm*U/2*(athermal1+athermal2) **(-1.0/2)
          hard2=h*exp(-k2*deqps) -(e0*k2*exp(-k2*deqps))
           hard=hard1*hard2
            write (*,*)'hard1=',hard1
             write (*,*)'hard2=',hard2
              write (*,*)'hard=', hard
C
C Find the new trace and stress tensor.
C
                  trace= strainInc(i,1) + strainInc(i,2) + strainInc(i,3)
                  s11= stressOld(i,1) + 2*G*strainInc(i,1) + alamda*trace
                  s22= stressOld(i,2) + 2*G*strainInc(i,2) + alamda*trace
                  s33= stressOld(i,3) + 2*G*strainInc(i,3) + alamda*trace
                  s12= stressOld(i,4) + 2*G*strainInc(i,4)
                  if (nshr.gt.1.0) then
                  s13= stressOld(i,5) + 2*G*strainInc(i,5)
                  s23= stressOld(i,6) + 2*G*strainInc(i,6)
                  end if
C Compute hydrostatic stress.
```

```
                  smean=(s11+s22+s33)/3.0
C
C Compute deviatoric stress from hydrostatic stress and stress tensor.
                  s1 = s11 - smean
                  s2 = s22 - smean
                  s3 = s33 - smean
                  s4 = s12
                  s5 = s13
                  s6 = s23
C Compute the equivalent mises stress from deviatoric stress.
               if (nshr.eq.1) then
                 vmises = sqrt(1.5*(s1**2+s2**2+s3**2+2*s4**2))
                else
               vmises= sqrt(1.5*(s1**2+s2**2+s3**2+2*(s4**2+s5**2+s6**2)))
                      write (*,*)'vmises=',vmises
                end if
C
                  sigdif = vmises - yieldOld
                  facyld = 0.0
                  if (sigdif.gt.0.0) facyld = 1.0
                  deqps = facyld*sigdif/(3*G + hard)
                  write (*,*)'deqps=',deqps
                  write (*,*)'dt=',dtArray(1)
                  write (*,*)'sigdif=',sigdif
C
C Compute the initial the plastic strain rate from increment in plastic
C strain.
                  if (deqps.gt.0.0) then
                            ddeqps=deqps/dtArray (1)
                        else
                            ddeqps=1.0
                  end if
                  write (*,*)'ddeqps=',ddeqps

C
C Update the stress
                  yieldNew = yieldOld + hard* deqps
                  write (*,*)'yieldNew=',yieldNew
                  factor   = yieldNew/(yieldNew+3*G*deqps)
                  write (*,*)'factor=', factor
C
C Find new stress tensor.
C
                  stressNew(i,1) = s1*factor + smean
                  stressNew(i,2) = s2*factor + smean
                  stressNew(i,3) = s3*factor + smean
                  stressNew(i,4) = s4*factor
                  if (nshr.gt.1) then
                    stressNew(i,5) = s5*factor
                    stressNew(i,6) = s5*factor
                 end if
C
C New equivalent deviatoric stress and von Mises equivalent
                  s1=s1*factor
                  s2=s2*factor
                  s3=s3*factor
                  s4=s4*factor
```

```
                  s5=s5*factor
                  s6=s6*factor
C Update the state variables.
                  stateNew(i,1) = stateOld(i,1) + deqps
                  stateNew(i,2) = ddeqps
                  stateNew(i,3) = yieldOld
                  write(*,*)'stateNew(i,1)=', stateNew(i,1)
                  write(*,*)'stateNew(i,3)=', stateNew(i,3)
C
C Update the specific internal energy.
C
                  sp1=(stressOld(i,1) +stressNew(i,1)) *strainInc(i,1)
                  sp2=(stressOld(i,2) +stressNew(i,2)) *strainInc(i,2)
                  sp3=(stressOld(i,3) +stressNew(i,3)) *strainInc(i,3)
                  sp4=(stressOld(i,4) +stressNew(i,4)) *strainInc(i,4)
                  sp5=(stressOld(i,5) +stressNew(i,5)) *strainInc(i,5)
                  sp6=(stressOld(i,6) +stressNew(i,6)) *strainInc(i,6)
           if (nshr.eq.1) then
        stress Power =0.5*(sp1+sp2+sp3) +sp4
         else
         stress Power = 0.5*(sp1+sp2+sp3) +sp4+sp5+sp6
           end if
         enerInternNew(i) = enerInternOld(i)+ stressPower / density(i)
C
C Update the dissipated inelastic energy.
C Transfer old value of inelastic specific energy
C         enerInelasNew(i) = enerInelasOld(i)
C
           plasticWorkInc=0.5*(yieldNew)*deqps
           write(*,*)'plasticWorkInc=', plasticWorkInc
           enerInelasNew(i)=enerInelasOld(i)+plasticWorkInc/density(i)

                  end do
          end if
      return
      end
```
